# Causal association between cholecystectomy and fracture: A Mendelian randomization study

**DOI:** 10.1097/MD.0000000000040795

**Published:** 2024-12-06

**Authors:** Shijie Zheng, Xinhui Xie

**Affiliations:** a Department of Spine Surgery, ZhongDa Hospital, School of Medicine, Southeast University, Nanjing, China.

**Keywords:** bone, cholecystectomy, fractures, genome-wide association study, Mendelian randomization analysis

## Abstract

Previous observational studies have reported that cholecystectomy is associated with an increased risk of fracture. However, the causality of this association remains unclear. This study aimed to explore the causal relationship between cholecystectomy and fracture using a Mendelian randomization (MR) approach. Our primary analytical method was the comprehensive two-sample MR analysis, with inverse variable weighting (IVW) serving as the main analysis technique. In addition, we use Bayesian weighted MR analysis to further confirm the results of IVW method. To enhance the robustness of our findings, we employed multiple analytical approaches including MR-Egger, weighted mode, simple mode, and weighted median. We further conducted sensitivity analyses to validate the stability and feasibility of our dataset. The results of IVW methods showed that there had no significant causal effect of cholecystectomy on fracture (forward *P* value: .82, .63, .96, .60, .19, .40, .58, .38, .37, .97, and .50 for fracture of wrist and hand, fracture of femur, fracture of foot, fracture of forearm, fracture of lower leg, fracture of lumbar spine and pelvis, fracture of neck, fracture of ribs, fracture of shoulder and upper arm, fracture of skull and facial bones, and fracture of spine), the results of Bayesian weighted MR showed similar results (*P* > .05). In the reverse, fracture of femur (*P* = .01) and fracture of shoulder and upper arm (*P* = .01) showed increased risks of cholecystectomy. The sensitivity analysis showed that none of our analyses were horizontally pleiotropic (*P* > .05 for MR-Egger’s intercept method). Our results do not support the causal effect of cholecystectomy on fracture, which was opposite to most previous observational studies.

## 1. Introduction

In the 2020 World Society of Emergency Surgery updated guidelines, cholecystectomy was recommended as the therapeutic approach for multiple gallbladder diseases, including gallstone, gallbladder polypoid lesions, and acute cholecystitis.^[1]^ With advanced surgical methods in recent years, the gallbladder can be successfully removed without surgical complications such as duct injury, bile leak, and vessel injury.^[2–4]^ Although cholecystectomy is viewed as a simple benign operation with no significant long-term effects on a patient’s health, it is associated with significant physiological changes in digestion and metabolism. Previous studies have shown that cholecystectomy is associated with an increased risk of diseases such as colon cancer, metabolic syndrome, and nonalcoholic fatty liver disease.^[5–8]^

Fracture is a very common disease, especially among the elderly, and it is considered a major health problem worldwide.^[9,10]^ Osteoporosis and vitamin D deficiency are considered risk factors for fractures.^[11,12]^ Previous studies have shown reduced vitamin levels and bone marrow density in patients undergoing cholecystectomy.^[13,14]^ Therefore, there may be an increased risk of fracture in patients after cholecystectomy. However, few studies explore the association between cholecystectomy and fracture. Lee et al^[15]^ demonstrated an increased risk of fracture replacement after cholecystectomy in a Korean cohort study. Similarly, an observational study suggested that patients who undergo cholecystectomy have a higher risk of developing osteoporosis later in life.^[16]^ However, another study suggests that the increased risk of fracture due to gallstone disease is not caused by cholecystectomy.^[17]^ Therefore, it is not clear whether there is an association between cholecystectomy and fracture.

Mendelian randomization (MR) study has a more robust confounding than observational studies, which has been proven to be a powerful approach for clarifying causal relationships using single nucleotide polymorphism (SNP) as instrumental variables (IVs).^[18]^ Because genetic variants are randomly separated during meiosis and are fixed throughout life, MR can minimize bias due to unmeasured confounders and reverse causation.^[18]^ Recently, some studies have used MR to explore the association between cholecystectomy and other diseases such as colorectal cancer, kidney cancer, and gastroesophageal reflux disease.^[19–21]^ As far as we know, no MR study has explored the association between cholecystectomy and fracture. This study aimed to explore the bidirectional causal relationship between cholecystectomy and fracture to facilitate the management and guidance of patients requiring cholecystectomy clinically.

## 2. Materials and methods

**Figure 1.**
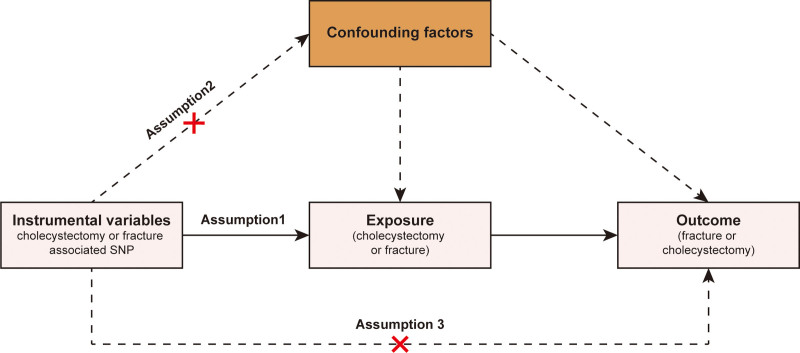
The flowchart of this MR study. MR = Mendelian randomization.

In this study, we performed a 2-sample MR to identify the potential association between cholecystectomy and 11 fractures including fracture of wrist and hand, fracture of femur, fracture of foot, fracture of forearm, fracture of lower leg, fracture of lumbar spine and pelvis, fracture of neck, fracture of ribs, fracture of shoulder and upper arm, fracture of skull and facial bones, and fracture of spine. The MR studies need to satisfy 3 core assumptions.^[22]^ First, the genetic IVs must be associated with exposure. Second, there is no confounding between genetic IVs and outcomes. Third, all association genetic IVs and outcomes should be fully mediated by the exposure. Figure 1 shows the flowchart of this study.

### 2.1. Data source

Our study aimed to explore the causal associations between cholecystectomy and 11 subtypes of fracture. The genome-wide association study (GWAS) summary statistical data for cholecystectomy (n = 463,010, comprised 10,361 cases and 452,649 controls) with European ancestry were downloaded from the public website “OPEN GWAS” (https://gwas.mrcieu.ac.uk/). The GWAS summary statistical data for fracture with European ancestry were obtained from FinnGen consortium (https://www.finngen.fi/en),^[23]^ including fracture of wrist and hand (n = 415,859, comprised 14,734 cases and 401,125 controls), fracture of femur (n = 443,891, comprised 10,714 cases and 433,177 controls), fracture of foot (n = 429,894, comprised 9782 cases and 420,112 controls), fracture of forearm (n = 445,339, comprised 24,647 cases and 420,692 controls), fracture of lower leg (n = 410,380, comprised 24,999 cases and 385,381 controls), fracture of lumbar spine and pelvis (n = 445,257, comprised 7867 cases and 437,390 controls), fracture of neck (n = 446,488, comprised 1859 cases and 444,629 controls), fracture of ribs (n = 446,295, comprised 11,410 cases and 434,885 controls), fracture of shoulder and upper arm (n = 427,937, comprised 14,824 cases and 413,113 controls), fracture of skull and facial bones (n = 401,123, comprised 8618 cases and 392,505 controls), and fracture of spine (n = 450,456, comprised 56 cases and 450,400 controls). The FinnGen study is a large-scale genomics initiative that has analyzed over 500,000 Finnish biobank samples and correlated genetic variation with health data to understand disease mechanisms and predispositions. The project is a collaboration between research organizations and biobanks within Finland and international industry partners. Table 1 provides the characteristics of those GWAS data. Since each study contributing to this MR research had already acquired the needed consent and ethical approval, there was no need for any further consent or ethical approval specifically for the current study.

**Table 1:** Characteristics of the GWAS dataset.

Variable	GWAS ID	Cases	Control	N
Cholecystectomy	ukb-b-13803	10,361	452,649	463,010
Fracture of wrist and hand	finngen_R11_ST19_FRACT_WRIST_HAND_LEVEL	14,734	401,125	415,859
Fracture of femur	finngen_R11_ST19_FRACT_FEMUR	10,714	433,177	443,891
Fracture of foot	finngen_R11_ST19_FRACT_FOOT_ANKLE	9782	420,112	429,894
Fracture of forearm	finngen_R11_ST19_FRACT_FOREA	24,647	420,692	445,339
Fracture of lower leg	finngen_R11_ST19_FRACT_LOWER_LEG_INCLU_ANKLE	24,999	385,381	410,380
Fracture of lumbar spine and pelvis	finngen_R11_ST19_FRACT_LUMBAR_SPINE_PELVIS	7867	437,390	445,257
Fracture of neck	finngen_R11_ST19_FRACT_NECK	1859	444,629	446,488
Fracture of ribs	finngen_R11_ST19_FRACT_RIBS_STERNUM_THORACIC_SPINE	11,410	434,885	446,295
Fracture of shoulder and upper arm	finngen_R11_ST19_FRACT_SHOUL_UPPER_ARM	14,824	413,113	427,937
Fracture of skull and facial bones	finngen_R11_ST19_FRACT_SKULL_FACIAL_BONES	8618	392,505	401,123
Fracture of spine	finngen_R11_ST19_FRACT_SPINE_LEVEL_UNSPE	56	450,400	450,456

GWAS = genome-wide association study.

### 2.2. Selection of IVs

This study satisfied 3 core assumptions of the MR study. To single out the IVs, we first extracted the SNPs robustly associated with the exposures (*P* < 5 × 10^−8^) in the forward MR. In the reverse MR analysis, we extended the threshold to *P* < 5 × 10^−6^. Then, we only retained independent SNPs (kb = 10,000, *r*^2^ < 0.001) based on the linkage disequilibrium structure of European populations. Finally, we used *F* statistics to assess the strength of those IVs. All IVs used in our MR analysis were gathered *F* > 10 to minimize the potential weak instrument bias.^[24]^ To avoid the influence of confounding factors, we excluded common confounders of AS such as weight, diabetes, and thyroid diseases by using the “FastTraitR” package.

### 2.3. Statistical analysis

All analyses were performed in R 4.3.3 software (https://www.r-project.org). We applied the “TwoSampleMR” package for the causal estimates.^[25]^ To evaluate the causal association between cholecystectomy and fracture, 5 MR analysis methods including inverse variance weighted (IVW), weighted median, weighted mode, simple mode, and MR-Egger regression test were performed. Among them, IVW was the main analysis method, and other analysis methods were auxiliary methods. In addition, we employed Bayesian weighted Mendelian randomization (BWMR) to further strengthen the reliability of IVW results. Then, a series of sensitivity analyses were performed. Cochran *Q* test quantified the heterogeneity of IVs, and *P* < .05 indicated heterogeneity. The MR-Egger method is used to exclude the influence of horizontal pleiotropy. If the intercept term is significant (*P* < .05), it indicates the existence of horizontal pleiotropy. Finally, we adopted Mendelian Randomization Pleiotropy Residual Sum and Outlier to identify outliers. If there is an outlier, we will remove the outlier and redo the above analysis. Among them, all results were presented as odds ratio (OR) and 95% confidence interval (CI). Finally, scatter plots, funnel plots, and leave-one-out analysis were performed. The scatter plot showed that the results were unaffected by outliers. The funnel plot showed the robustness of the correlation without heterogeneity. The leave-one-out sensitivity test was used to evaluate whether causal effects are significantly influenced by a single SNP.

## 3. Results

### 3.1. Instrument variable

In the bidirectional 2-sample Mendelian randomization analysis, we screened for IVs from exposure data. All IVs had *F* value >10, suggesting no weak instrument bias in the present study. Supplementary File 1, Supplemental Digital Content, http://links.lww.com/MD/O110, provides detailed information on IVs screened for all positive results.

### 3.2. Forward analysis of cholecystectomy on fracture

**Figure 2.**
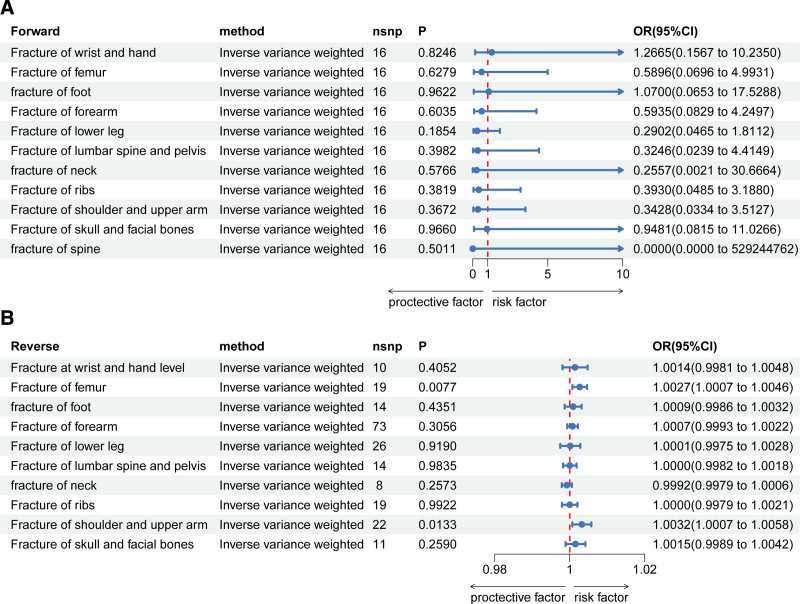
MR analysis had no significant effect of cholecystectomy on fracture. (A) Forward MR analysis of cholecystectomy on fracture. (B) Reverse MR analysis of fracture on cholecystectomy. CI = confidence interval, MR = Mendelian randomization, nsnp = number of available SNP, OR = odds ratio.

**Figure 3.**
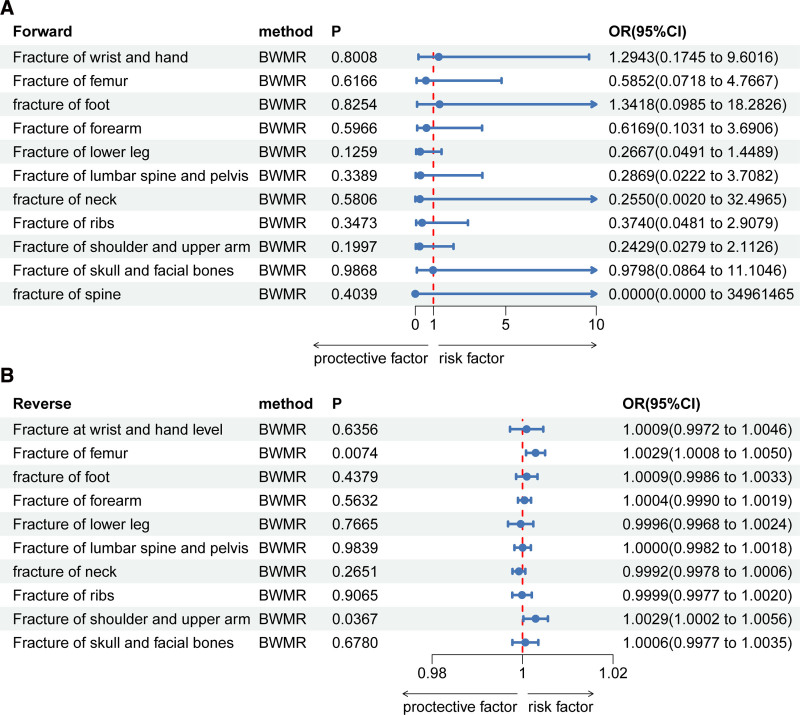
The results of BWMR analysis showed a similar trend with IVW method. (A) Forward BWMR analysis of cholecystectomy on fracture. (B) Reverse BWMR analysis of fracture on cholecystectomy. CI = confidence interval, BWMR = Bayesian weighted Mendelian randomization, OR = odds ratio.

Figure 2A shows the forest plots of IVW results of forward MR analysis of cholecystectomy on fracture. In the mainly IVW methods, genetically predicted cholecystectomy was not associated with the risk of fracture (*P* > .05). The results of BWMR were the same as those of IVW (*P* > .05) (Fig. 3A). All detailed information of forward MR analysis including MR-Egger, weighted median, simple mode, and weighted mode could be found in Supplementary File 2, Supplemental Digital Content, http://links.lww.com/MD/O111. Our results showed that no causal effect existed for cholecystectomy on fracture. The scatter plots of forward MR analysis are provided in Supplementary Figure 1, Supplemental Digital Content, http://links.lww.com/MD/O112.

### 3.3. Reverse analysis of fracture on cholecystectomy

Figure 2B shows the forest plots of IVW results of reverse MR analysis of fracture on cholecystectomy. Since there were still no positive IVs in the fracture of spine at a threshold of *P* = 5 × 10^−5^, we concluded that the fracture of spine had no effect on cholecystectomy. The results of IVW analysis showed that fracture of femur (*P* = .008, OR = 1.003) and fracture of shoulder and upper arm (*P* = .013, OR = 1.003) had an increased risk of cholecystectomy. Other fractures had no causal effect on cholecystectomy (*P* > .05), and the detailed information of MR-Egger, weighted median, simple mode, and weighted mode are shown in Supplementary File 2, Supplemental Digital Content, http://links.lww.com/MD/O111. The results of BWMR analysis showed the same trend as those of IVW analysis (Fig. 3B). The scatter plots are shown in Supplementary Figure 2, Supplemental Digital Content, http://links.lww.com/MD/O118.

### 3.4. Sensitivity analysis

In the sensitivity analysis of the Cochran *Q* test (Table 2), though fracture of foot (*P* = .03), fracture of forearm (*P* = .01), fracture of lower leg (*P* = .02), and fracture of shoulder and upper arm (*P* = .03) showed heterogeneity in the forward MR analysis, our results were still credible. Besides, the sensitivity analysis of MR-Egger method showed that none of the above MR analyses were horizontally pleiotropic (Table 3, *P* > .05 for MR-Egger’s intercept method), which further proved that our results were credible. The plots of leave-one-out method (Supplementary Figure 3, Supplemental Digital Content, http://links.lww.com/MD/O121, Supplementary Figure 4, Supplemental Digital Content, http://links.lww.com/MD/O120) and funnel (Supplementary Figure 5, Supplemental Digital Content, http://links.lww.com/MD/O122, Supplementary Figure 6, Supplemental Digital Content, http://links.lww.com/MD/O123) showed stable and reliable data. Finally, the Mendelian Randomization Pleiotropy Residual Sum and Outlier test showed that there had no outlier was detected.

**Table 2:** Cochran *Q* test for heterogeneity.

	Cochran *Q* test		
Exposure/outcome	Method	Cochran *Q*	*P*
Fracture of wrist and hand	IVW (forward reverse)	21.85 8.24	.11 .51
Fracture of femur	IVW (forward reverse)	16.14 16.93	.37 .53
Fracture of foot	IVW (forward reverse)	26.40 11.75	.03 .55
Fracture of forearm	IVW (forward reverse)	31.56 77.65	.01 .30
Fracture of lower leg	IVW (forward reverse)	27.88 30.44	.02 .19
Fracture of lumbar spine and pelvis	IVW (forward reverse)	18.43 6.84	.24 .91
Fracture of neck	IVW (forward reverse)	13.84 5.59	.54 .59
Fracture of ribs	IVW (forward reverse)	17.06 18.86	.32 .40
Fracture of shoulder and upper arm	IVW (forward reverse)	27.35 28.50	.03 .13
Fracture of skull and facial bones	IVW (forward reverse)	17.93 14.00	.27 .17
Fracture of spine	IVW (forward reverse)	18.77 NA	.22 NA

IVW = inverse variable weighting.

**Table 3:** MR-Egger intercept test for horizontal pleiotropy.

		MR-Egger intercept test		
Exposure/outcome		Egger intercept	SE	*P*
Fracture of wrist and hand	Forward Reverse	−0.00003 −0.00073	0.00679 0.00047	0.99638 0.15805
Fracture of femur	Forward Reverse	0.00492 0.00047	0.00680 0.00026	0.48113 0.08889
fracture of foot	Forward Reverse	−0.00653 0.00008	0.00891 0.00044	0.47557 0.85180
Fracture of forearm	Forward Reverse	0.00184 −0.00007	0.00637 0.00015	0.77615 0.61833
Fracture of lower leg	Forward Reverse	0.00319 0.00017	0.00589 0.00021	0.59650 0.42418
Fracture of lumbar spine and pelvis	Forward Reverse	0.01123 −0.00010	0.00793 0.00031	0.17858 0.74529
Fracture of neck	Forward Reverse	−0.00377 0.00015	0.01502 0.00051	0.80533 0.77338
Fracture of ribs	Forward Reverse	0.00573 0.00028	0.00662 0.00026	0.40155 0.30269
Fracture of shoulder and upper arm	Forward Reverse	0.01374 0.00025	0.00661 0.00050	0.05638 0.61747
Fracture of skull and facial bones	Forward Reverse	−0.00981 0.00070	0.00753 0.00032	0.21372 0.05481
Fracture of spine	Forward Reverse	0.13701 NA	0.09226 NA	0.15969 NA

MR = Mendelian randomization.

## 4. Discussion

In this study, we first explore the causal relationship between cholecystectomy and fracture using MR analysis. Totally, 12 GWAS datasets were incorporated into our study, including cholecystectomy GWAS dataset (ukb-b-6235) and 11 fracture GWAS datasets (fracture of wrist and hand, fracture of femur, fracture of foot, fracture of forearm, fracture of lower leg, fracture of lumbar spine and pelvis, fracture of neck, fracture of ribs, fracture of shoulder and upper arm, fracture of skull and facial bones, and fracture of spin). Our MR analysis indicated that there was no causal effect of cholecystectomy on fracture.

Previous studies have investigated the association between cholecystectomy and the risk of fracture/osteoporosis, but the results are inconsistent. The mechanism of fracture risk after cholecystectomy is not well understood. In addition to changes in vitamin D synthesis and metabolism, it may also be related to bile, gut microbiota, and the immune system.^[26–28]^ Studies have shown that cholecystectomy is associated with vitamin D levels, and vitamin D is one of the factors that lead to osteoporosis.^[13,14,29,30]^ Therefore, cholecystectomy may play an important role in fracture risk. The first study to investigate fracture rates in participants undergoing cholecystectomy included individuals who underwent cholecystectomy (n = 143,667) and controls (n = 255,522) in the Korean population between 2010 and 2015, verified that people who undergo cholecystectomy have an increased risk of fracture after matching by age and sex.^[15]^ On the contrary, some studies indicated no association between cholecystectomy and fracture risk.^[17,31]^ These differences in results may be due to population differences and confounding factors present in observational studies. In a recent prospective study using data from the UK Biobank, they found that cholecystectomy was associated with a higher risk of osteoporosis in women (hazard ratio = 1.21, 95% CI = 1.12–1.31, *P* < .001) and men (hazard ratio = 1.45, 95% CI = 1.10–1.90, *P* = .007), and the association between cholecystectomy and osteoporosis was stronger in men than in women.^[16]^ This may be related to sex-specific differences in sex hormones and bone biology and morphology.

These studies cannot answer the question of causation. Therefore, our study aimed to explore the causal association between cholecystectomy and fracture using an MR approach, which could avoid the weakness of producing a relatively accurate causal judgment. In our study, we used GWAS data from cholecystectomies and 11 fractures including fracture of wrist and hand, fracture of femur, fracture of foot, fracture of forearm, fracture of lower leg, fracture of lumbar spine and pelvis, fracture of neck, fracture of ribs, fracture of shoulder and upper arm, fracture of skull and facial bones, and fracture of spin. In the forward MR analysis of the IVW method, there was no significant causal association. Interestingly, this is the opposite of all previous results. Other MR analysis methods including weighted median, weighted mode, simple mode, and MR-Egger showed a similar trend to IVW. The results of previous observational studies may be due to reverse causality. Our study confirmed that there was no significant genetic underlying association of cholecystectomy on fracture.

There are several advantages to our MR study. First, as we know, this is the first study to explore the causal relationship between cholecystectomy and fracture. Compared with previous observational studies, our MR study could exclude potential bias such as confounders and reverse causation, thus having higher statistical efficiency. Second, the GWAS datasets used in our study were based on European ancestry, and have a large number of individuals. The results are more reliable and stable. Finally, we performed multiple methods for analysis and sensitivity analysis to verify the reliability of the results.

However, there are limitations to our study. First, the study’s population is European and cannot be extended to other ethnic groups. Second, although we have adopted various methods to avoid horizontal pleiotropy, the existence of horizontal pleiotropy cannot be completely ruled out. Third, due to the lack of personal information, we were unable to conduct further stratified analysis of the population. Finally, larger sample sizes and more advanced methods are needed to corroborate the results, and more prospective studies are needed to prove our point.

## 5. Conclusion

Our 2-sample Mendelian randomization study indicated that there was no causal association of cholecystectomy on fracture, the association shown in previous observational studies may be due to unmeasured confounding or reverse causality.

## Acknowledgments

We acknowledge the participants and investigators of the FinnGen study and all the participants of the GWAS.

## Author contributions

**Data curation:** Shijie Zheng, Xinhui Xie.

**Formal analysis:** Shijie Zheng.

**Investigation:** Shijie Zheng.

**Methodology:** Shijie Zheng, Xinhui Xie.

**Resources:** Shijie Zheng, Xinhui Xie.

**Software:** Shijie Zheng, Xinhui Xie.

**Validation:** Shijie Zheng, Xinhui Xie.

**Visualization:** Shijie Zheng.

**Writing—original draft:** Shijie Zheng.

**Conceptualization:** Xinhui Xie.

**Project administration:** Xinhui Xie.

**Supervision:** Xinhui Xie.

**Writing—review & editing:** Xinhui Xie.

## Supplementary Material



## References

[1] Pisano M Allievi N Gurusamy K. 2020 World Society of Emergency Surgery updated guidelines for the diagnosis and treatment of acute calculus cholecystitis. World J Emerg Surg. 2020;15:61. 33153472 10.1186/s13017-020-00336-x PMC7643471

[2] Strasberg SM. A three-step conceptual roadmap for avoiding bile duct injury in laparoscopic cholecystectomy: an invited perspective review. J Hepatobiliary Pancreat Sci. 2019;26:123–7. 30828991 10.1002/jhbp.616

[3] Acar N Acar T Sur Y. Is subtotal cholecystectomy safe and feasible? Short- and long-term results. J Hepatobiliary Pancreat Sci. 2021;28:263–71. 33058478 10.1002/jhbp.847

[4] Sun N Zhang JL Zhang CS Li XH Shi Y. Single-incision robotic cholecystectomy versus single-incision laparoscopic cholecystectomy: a systematic review and meta-analysis. Medicine (Baltimore). 2018;97:e12103. 30200093 10.1097/MD.0000000000012103 PMC6133478

[5] Shen C Wu X Xu C Yu C Chen P Li Y. Association of cholecystectomy with metabolic syndrome in a Chinese population. PLoS One. 2014;9:e88189. 24505425 10.1371/journal.pone.0088189 PMC3914934

[6] Chen Y Wu S Tian Y. Cholecystectomy as a risk factor of metabolic syndrome: from epidemiologic clues to biochemical mechanisms. Lab Invest. 2018;98:7–14. 28892095 10.1038/labinvest.2017.95

[7] Zhang Y Liu H Li L. Cholecystectomy can increase the risk of colorectal cancer: a meta-analysis of 10 cohort studies. PLoS One. 2017;12:e0181852. 28771518 10.1371/journal.pone.0181852 PMC5542607

[8] Ruhl CE Everhart JE. Relationship of non-alcoholic fatty liver disease with cholecystectomy in the US population. Am J Gastroenterol. 2013;108:952–8. 23545713 10.1038/ajg.2013.70

[9] Cummings SR Melton LJ. Epidemiology and outcomes of osteoporotic fractures. Lancet. 2002;359:1761–7. 12049882 10.1016/S0140-6736(02)08657-9

[10] NIH Consensus Development Panel on Osteoporosis Prevention, Diagnosis, and Therapy. Osteoporosis prevention, diagnosis, and therapy. JAMA. 2001;285:785–95. 11176917 10.1001/jama.285.6.785

[11] Wang CY Fu SH Yang RS Shen LJ Wu FL Hsiao FY. Age- and gender-specific epidemiology, treatment patterns, and economic burden of osteoporosis and associated fracture in Taiwan between 2009 and 2013. Arch Osteoporos. 2017;12:92. 29067572 10.1007/s11657-017-0385-5

[12] Chakhtoura M Chamoun N Rahme M Fuleihan GE. Impact of vitamin D supplementation on falls and fractures-a critical appraisal of the quality of the evidence and an overview of the available guidelines. Bone. 2020;131:115112. 31676406 10.1016/j.bone.2019.115112

[13] Marcinowska-Suchowierska EB Talalaj MJ Wlodarcyzk AW Bielecki K Zawadzki JJ Brzozowski R. Calcium/phosphate/vitamin D homeostasis and bone mass in patients after gastrectomy, vagotomy, and cholecystectomy. World J Surg. 1995;19:597–601; discussion 601. 7676706 10.1007/BF00294730

[14] Polat HB Beyazal MS. The effect of cholecystectomy on 25-hydroxyvitamin D levels and bone mineral density in postmenopausal women. Arch Osteoporos. 2018;13:61. 29790021 10.1007/s11657-018-0458-0

[15] Lee EJ Shin CM Lee DH. The association between cholecystectomy and the risk for fracture: a nationwide population-based cohort study in Korea. Front Endocrinol (Lausanne). 2021;12:657488. 34122336 10.3389/fendo.2021.657488 PMC8190474

[16] Yang Q Wang M Zhang T Wen J Long L Xia C. Association of cholecystectomy with osteoporosis risk: a prospective study using data from the UK Biobank. Front Endocrinol (Lausanne). 2023;14:1259475. 37929032 10.3389/fendo.2023.1259475 PMC10623420

[17] Lin CJ Hsu CJ Chen CH. The association between gallstone disease (GSD) and hip fracture: a nationwide population-based study. Postgrad Med. 2021;133:357–61. 33337258 10.1080/00325481.2020.1866865

[18] Sanderson E Glymour MM Holmes MV. Mendelian randomization. Nat Rev Methods Primers. 2022;2:6. 37325194 10.1038/s43586-021-00092-5 PMC7614635

[19] Chen L Fan Z Sun X. Associations of cholecystectomy with the risk of colorectal cancer: a Mendelian randomization study. Chin Med J (Engl). 2023;136:840–7. 37027252 10.1097/CM9.0000000000002612 PMC10150870

[20] Qian J Xu H Liu J Zheng Y. Associations of cholecystectomy with the risk of gastroesophageal reflux disease: a Mendelian randomization study. Int J Surg. 2024;13:6836. 10.1097/JS9.0000000000001806 PMC11487017 38869973

[21] Kharazmi E Scherer D Boekstegers F. Gallstones, cholecystectomy, and kidney cancer: observational and Mendelian randomization results based on large cohorts. Gastroenterology. 2023;165:218–27.e8. 37054756 10.1053/j.gastro.2023.03.227

[22] Davies NM Holmes MV Davey SG. Reading Mendelian randomisation studies: a guide, glossary, and checklist for clinicians. BMJ. 2018;362:k601. 30002074 10.1136/bmj.k601 PMC6041728

[23] Kurki MI Karjalainen J Palta P; FinnGen. FinnGen provides genetic insights from a well-phenotyped isolated population. Nature. 2023;613:508–18. 36653562 10.1038/s41586-022-05473-8 PMC9849126

[24] Liu Z Wang H Yang Z Lu Y Zou C. Causal associations between type 1 diabetes mellitus and cardiovascular diseases: a Mendelian randomization study. Cardiovasc Diabetol. 2023;22:236. 37659996 10.1186/s12933-023-01974-6 PMC10475187

[25] Hemani G Zheng J Elsworth B. The MR-base platform supports systematic causal inference across the human phenome. Elife. 2018;7:e34408. 29846171 10.7554/eLife.34408 PMC5976434

[26] Di Ciaula A Garruti G Wang DQ Portincasa P. Cholecystectomy and risk of metabolic syndrome. Eur J Intern Med. 2018;53:3–11. 29706426 10.1016/j.ejim.2018.04.019 PMC8118133

[27] Wang W Wang J Li J. Cholecystectomy damages aging-associated intestinal microbiota construction. Front Microbiol. 2018;9:1402. 29988510 10.3389/fmicb.2018.01402 PMC6026649

[28] Sjogren K Engdahl C Henning P. The gut microbiota regulates bone mass in mice. J Bone Miner Res. 2012;27:1357–67. 22407806 10.1002/jbmr.1588 PMC3415623

[29] Ekiz T Yegen SF Katar MK Genc O Genc S. 25-Hydroxyvitamin D levels and bone mineral density evaluation in patients with cholecystectomy: a case-control study. Arch Osteoporos. 2018;13:14. 29500745 10.1007/s11657-018-0435-7

[30] Shabanzadeh DM Jorgensen T Linneberg A Sorensen LT Skaaby T. Vitamin D and gallstone disease-a population-based study. Endocrine. 2016;54:818–25. 27696253 10.1007/s12020-016-1113-4

[31] Nielsen CV Folkestad L Kroijer R Hansen SG. The risk of osteoporosis is not increased after cholecystectomy. A nationwide cohort study. Bone. 2023;173:116782. 37120083 10.1016/j.bone.2023.116782

