# Supplementary material for: Causal association between cholecystectomy and fracture: A Mendelian randomization study
Source: Medicine (Baltimore). 2024 Dec 6;103(49):e40795. doi: 10.1097/MD.0000000000040795 (PMC11630995; doi:10.1097/MD.0000000000040795)
Supplement: Supplementary file 3 [file medi-103-e40795-s003.pdf]

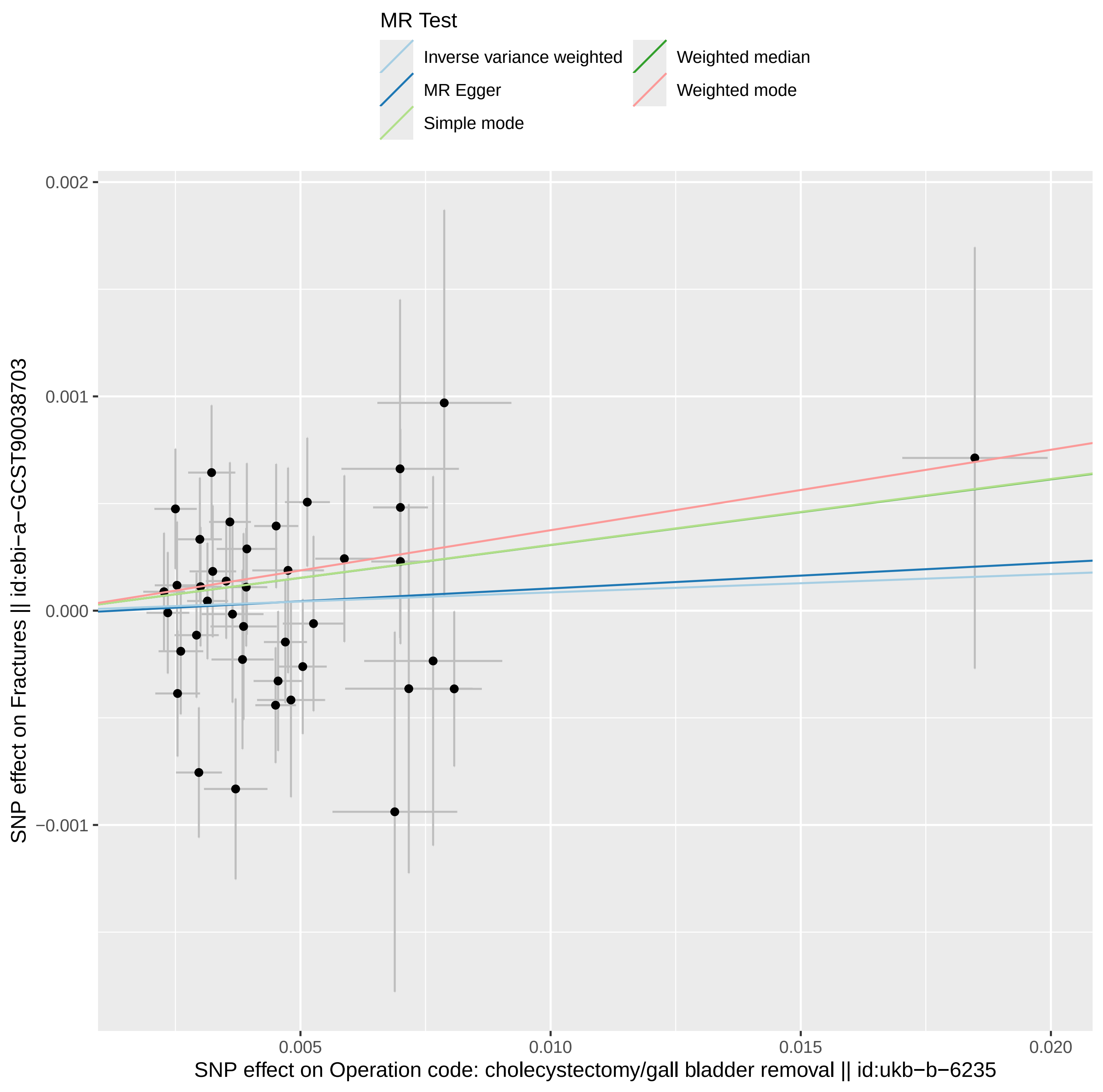

MR Test
Inverse variance weighted
MR Egger
Simple mode
Weighted median
Weighted mode
0.002
0.001
0.000
−0.001
0.005
0.010
0.015
0.020
SNP effect on Fractures || id:ebi−a−GCST90038703
SNP effect on Operation code: cholecystectomy/gall bladder removal || id:ukb−b−6235

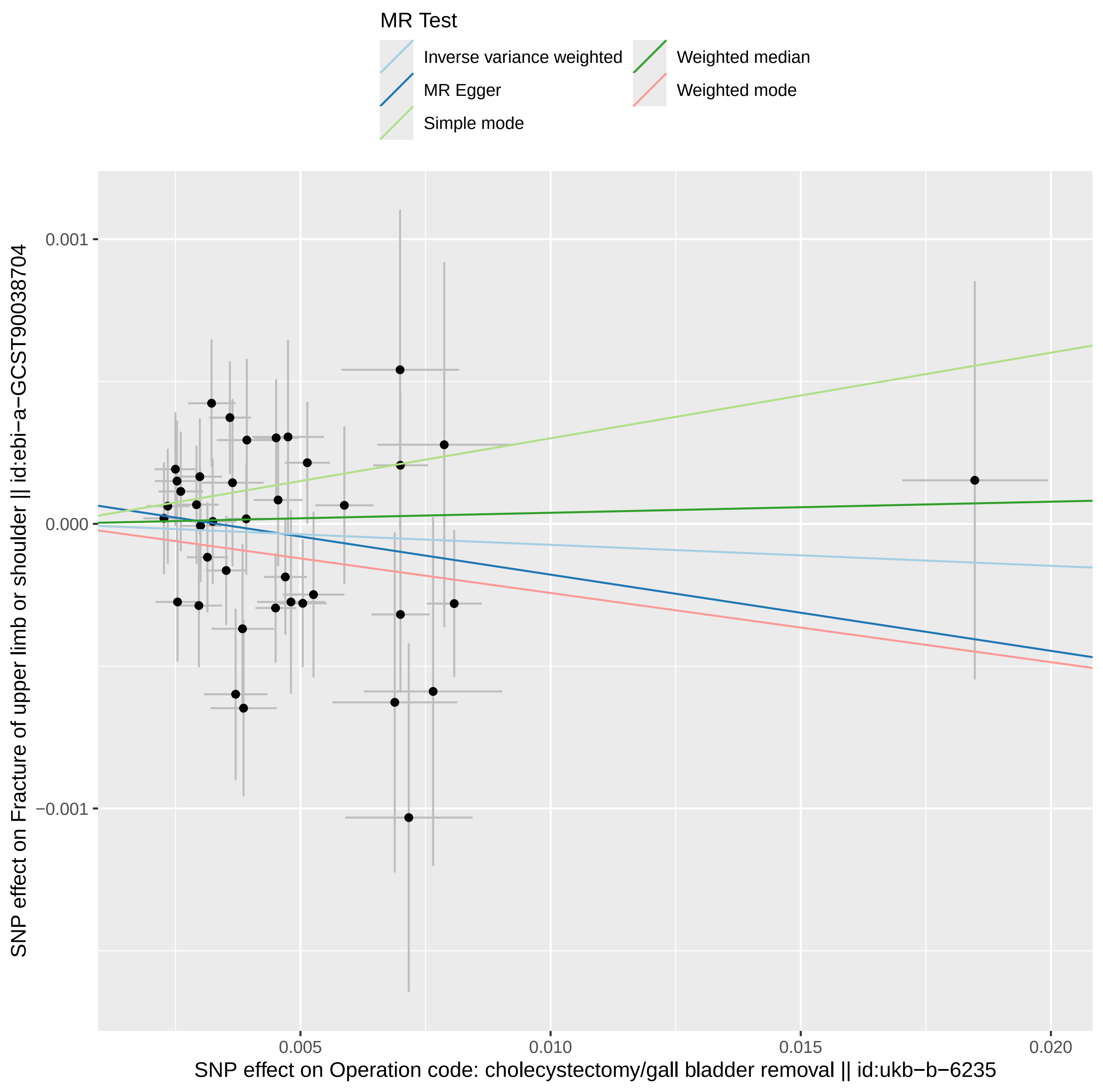
MR Test
Inverse variance weighted
MR Egger
Simple mode
Weighted median
Weighted mode
0.001
0.000
−0.001
0.005
0.010
0.015
0.020
SNP effect on Fracture of upper limb or shoulder || id:ebi−a−GCST90038704
SNP effect on Operation code: cholecystectomy/gall bladder removal || id:ukb−b−6235

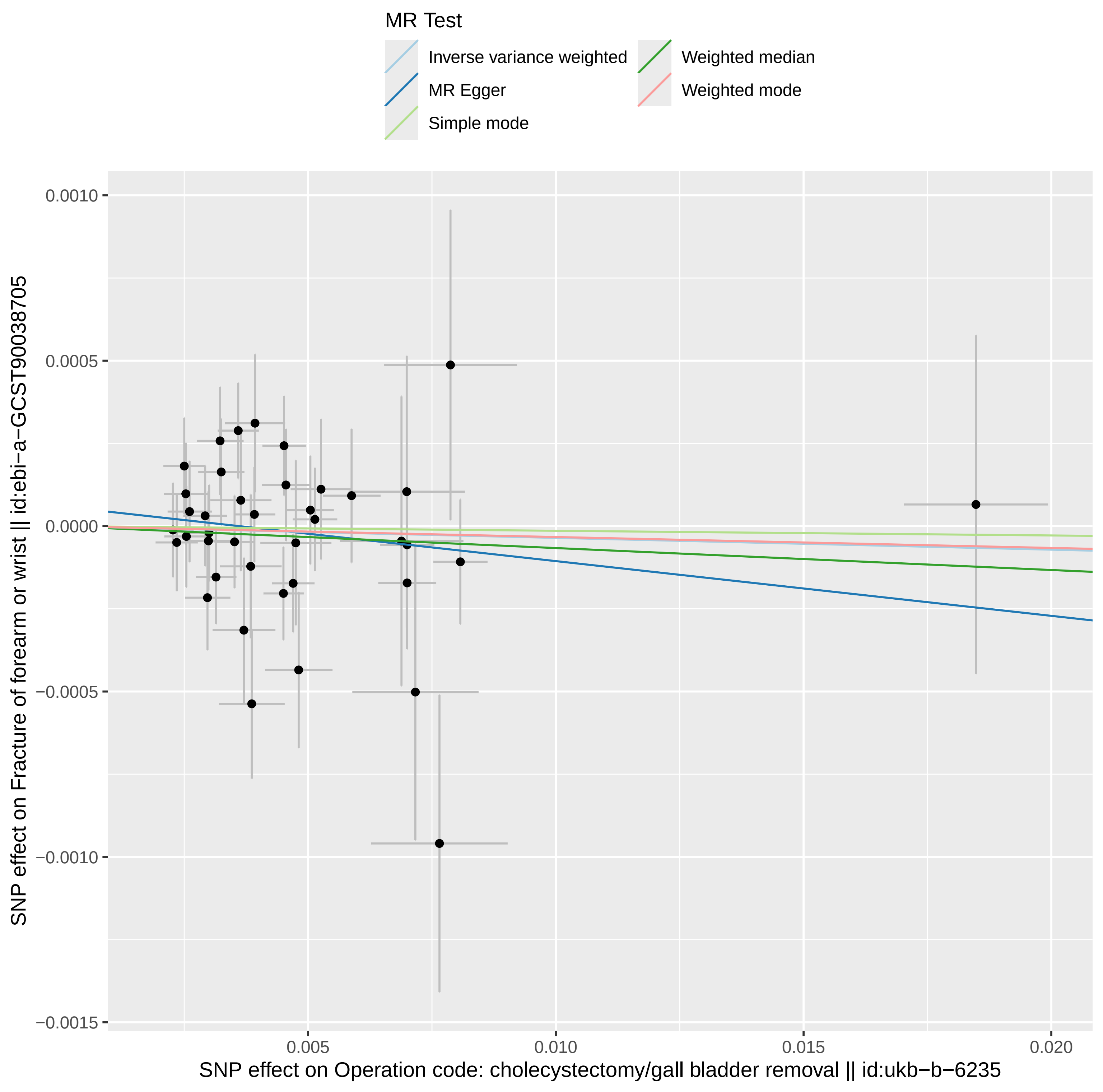

MR Test
Inverse variance weighted
MR Egger
Simple mode
Weighted median
Weighted mode
0.0010
0.0005
0.0000
−0.0005
−0.0010
−0.0015
0.005
0.010
0.015
0.020
SNP effect on Fracture of forearm or wrist || id:ebi−a−GCST90038705
SNP effect on Operation code: cholecystectomy/gall bladder removal || id:ukb−b−6235

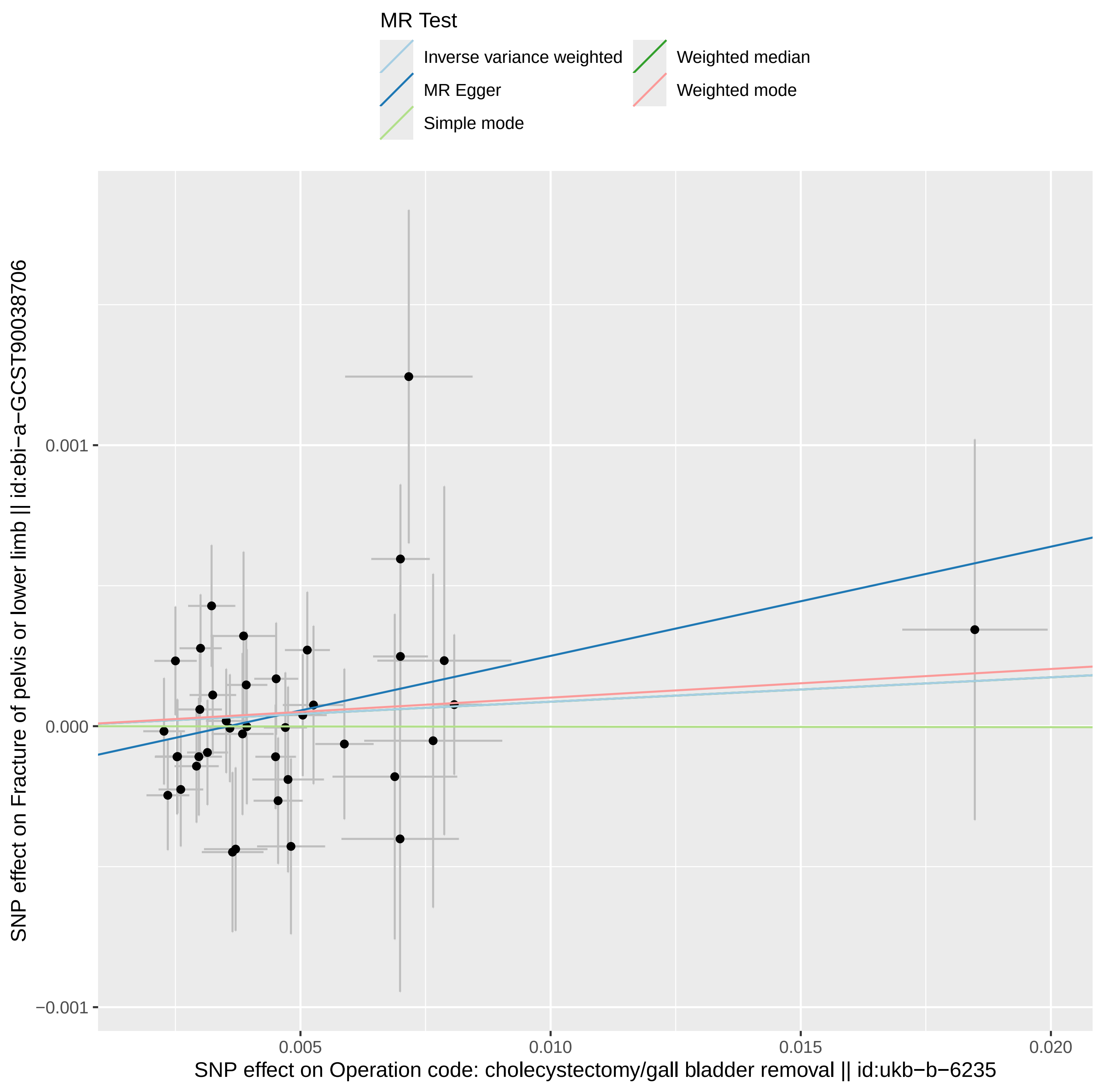

MR Test
Inverse variance weighted
MR Egger
Simple mode
Weighted median
Weighted mode
0.001
0.000
−0.001
0.005
0.010
0.015
0.020
SNP effect on Fracture of pelvis or lower limb || id:ebi−a−GCST90038706
SNP effect on Operation code: cholecystectomy/gall bladder removal || id:ukb−b−6235

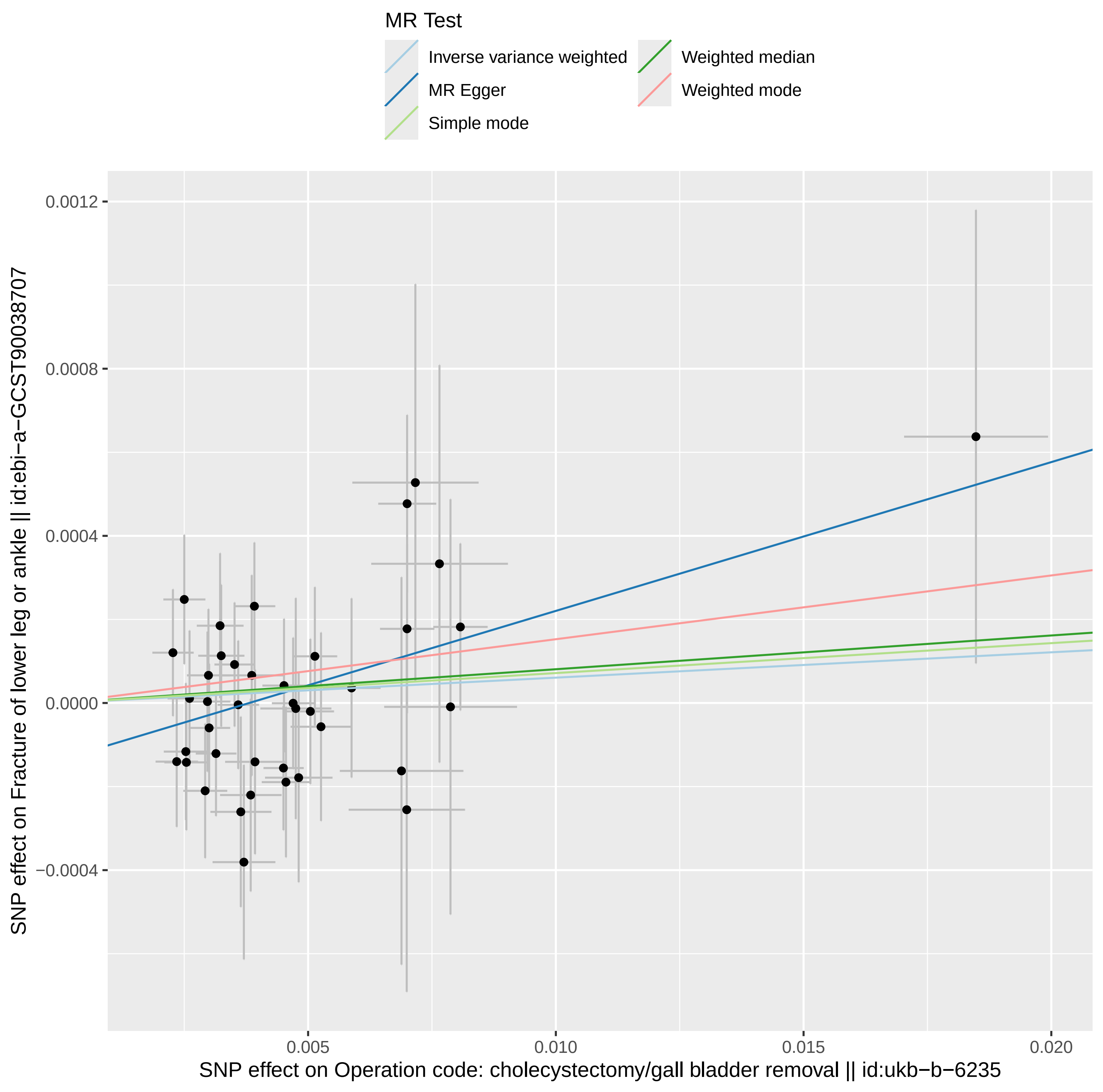
MR Test
Inverse variance weighted
MR Egger
Simple mode
Weighted median
Weighted mode
0.0012
0.0008
0.0004
0.0000
−0.0004
0.005
0.010
0.015
0.020
SNP effect on Operation code: cholecystectomy/gall bladder removal || id:ukb−b−6235
SNP effect on Fracture of lower leg or ankle || id:ebi−a−GCST90038707
