# Supplementary material for: Causal association between cholecystectomy and fracture: A Mendelian randomization study
Source: Medicine (Baltimore). 2024 Dec 6;103(49):e40795. doi: 10.1097/MD.0000000000040795 (PMC11630995; doi:10.1097/MD.0000000000040795)
Supplement: Supplementary file 4 [file medi-103-e40795-s004.pdf]

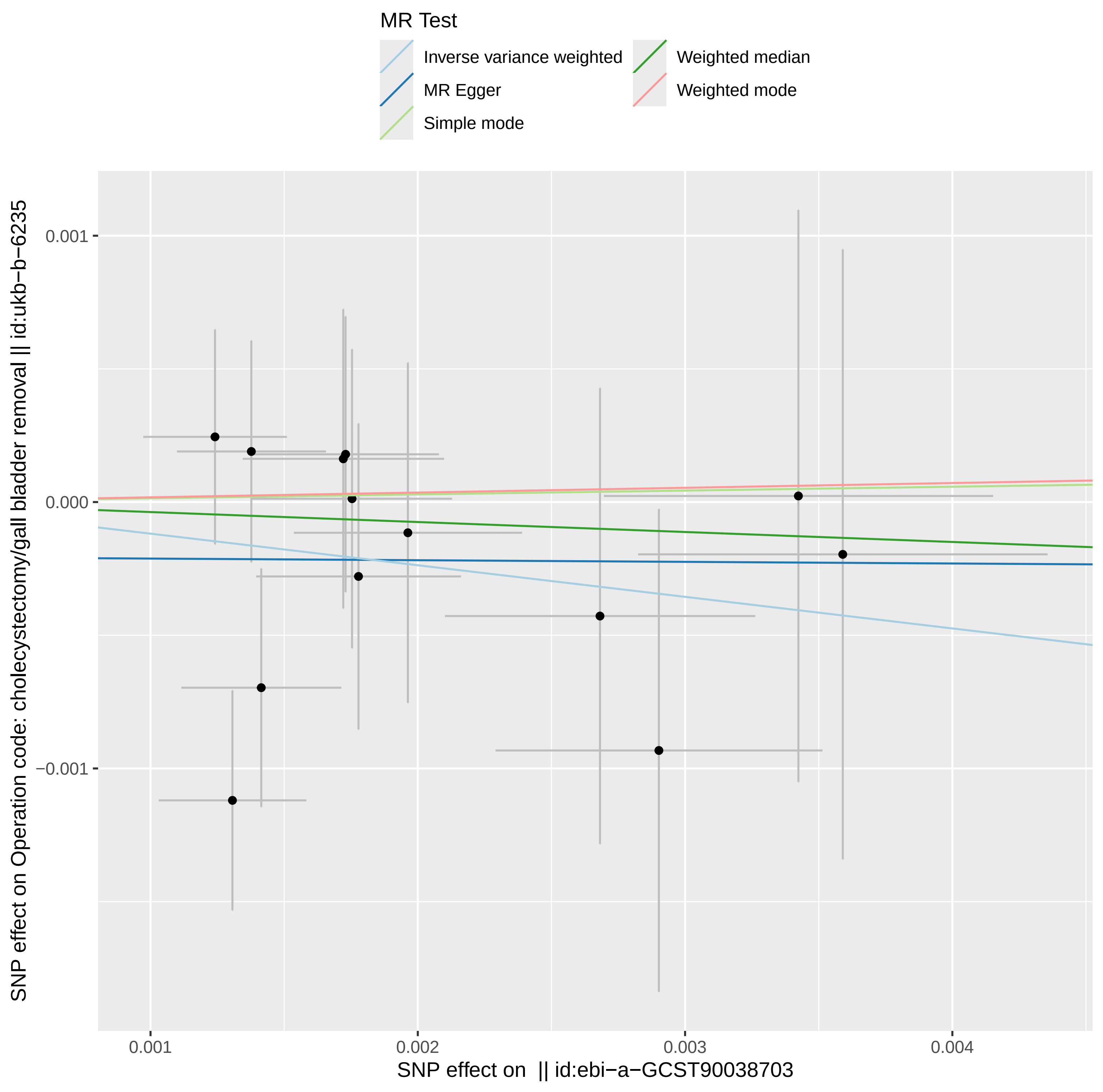

MR Test
Inverse variance weighted
MR Egger
Simple mode
Weighted median
Weighted mode
0.001
0.000
−0.001
0.001
0.002
0.003
0.004
SNP effect on || id:ebi−a−GCST90038703
SNP effect on Operation code: cholecystectomy/gall bladder removal || id:ukb−b−6235

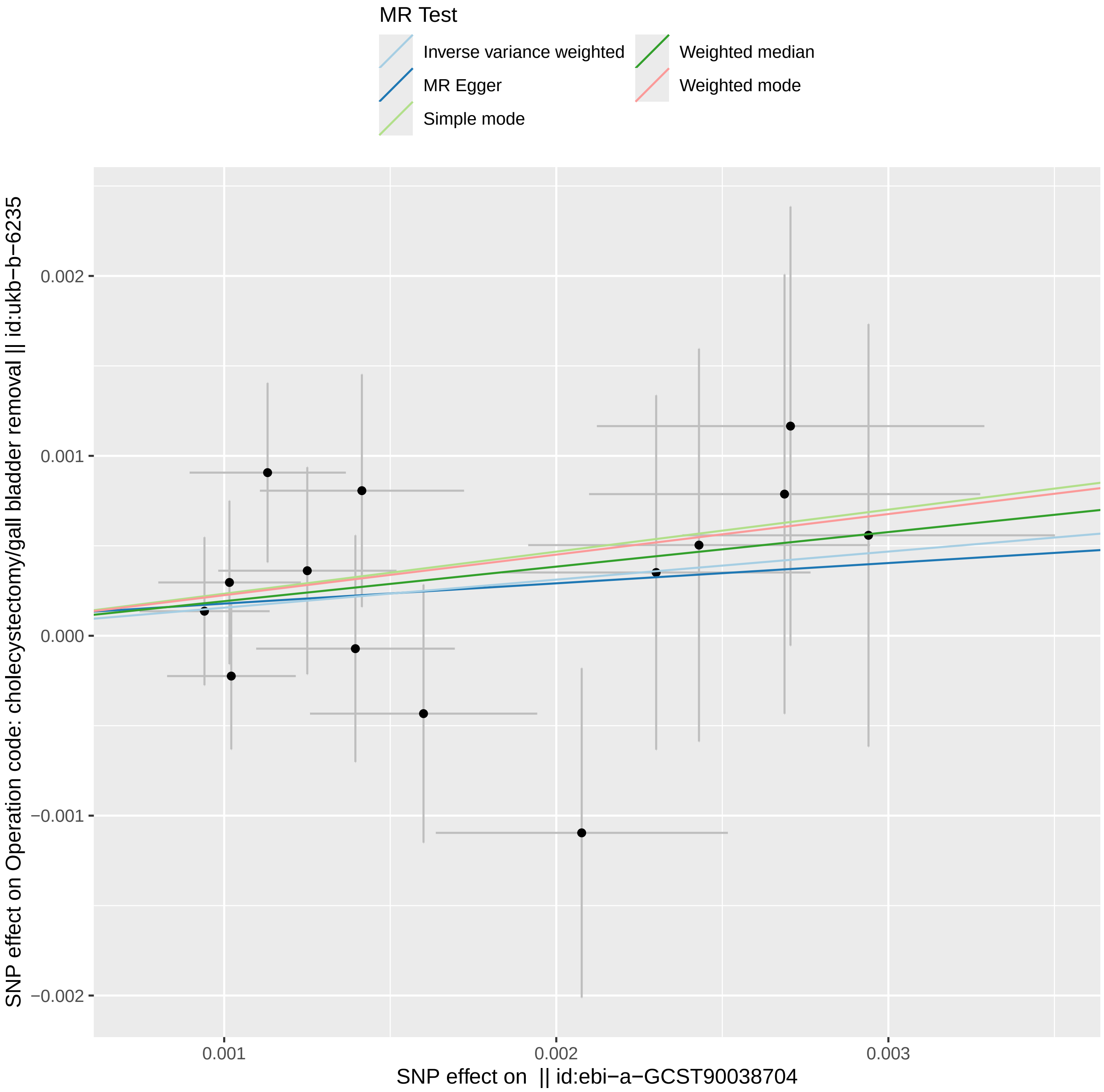
MR Test
Inverse variance weighted
MR Egger
Simple mode
Weighted median
Weighted mode
0.002
0.001
0.000
−0.001
−0.002
SNP effect on Operation code: cholecystectomy/gall bladder removal || id:ukb−b−6235
0.001
0.002
0.003
SNP effect on || id:ebi−a−GCST90038704

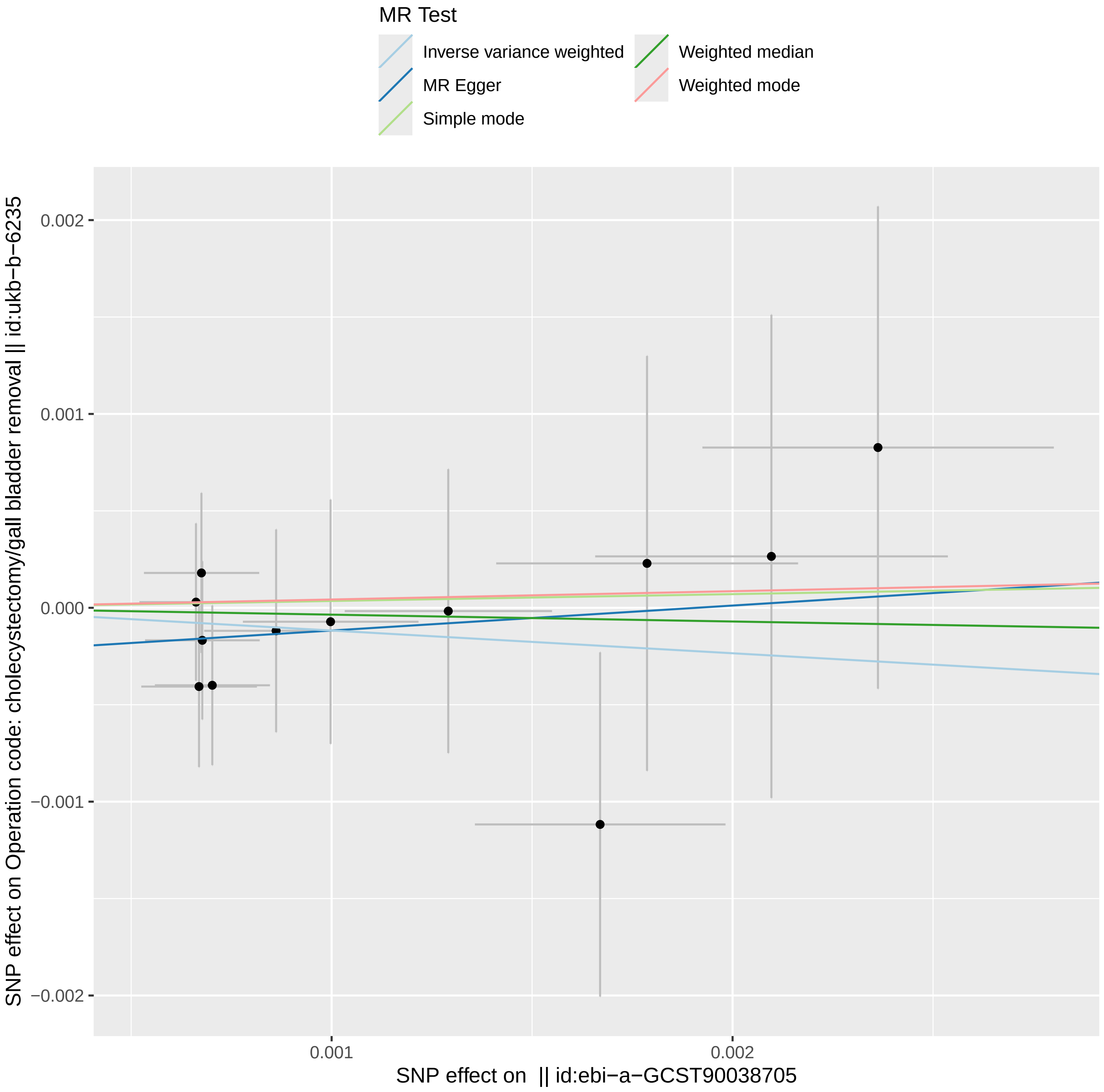
MR Test
Inverse variance weighted
MR Egger
Simple mode
Weighted median
Weighted mode
0.002
0.001
0.000
−0.001
−0.002
0.001
0.002
SNP effect on Operation code: cholecystectomy/gall bladder removal || id:ukb−b−6235
SNP effect on || id:ebi−a−GCST90038705

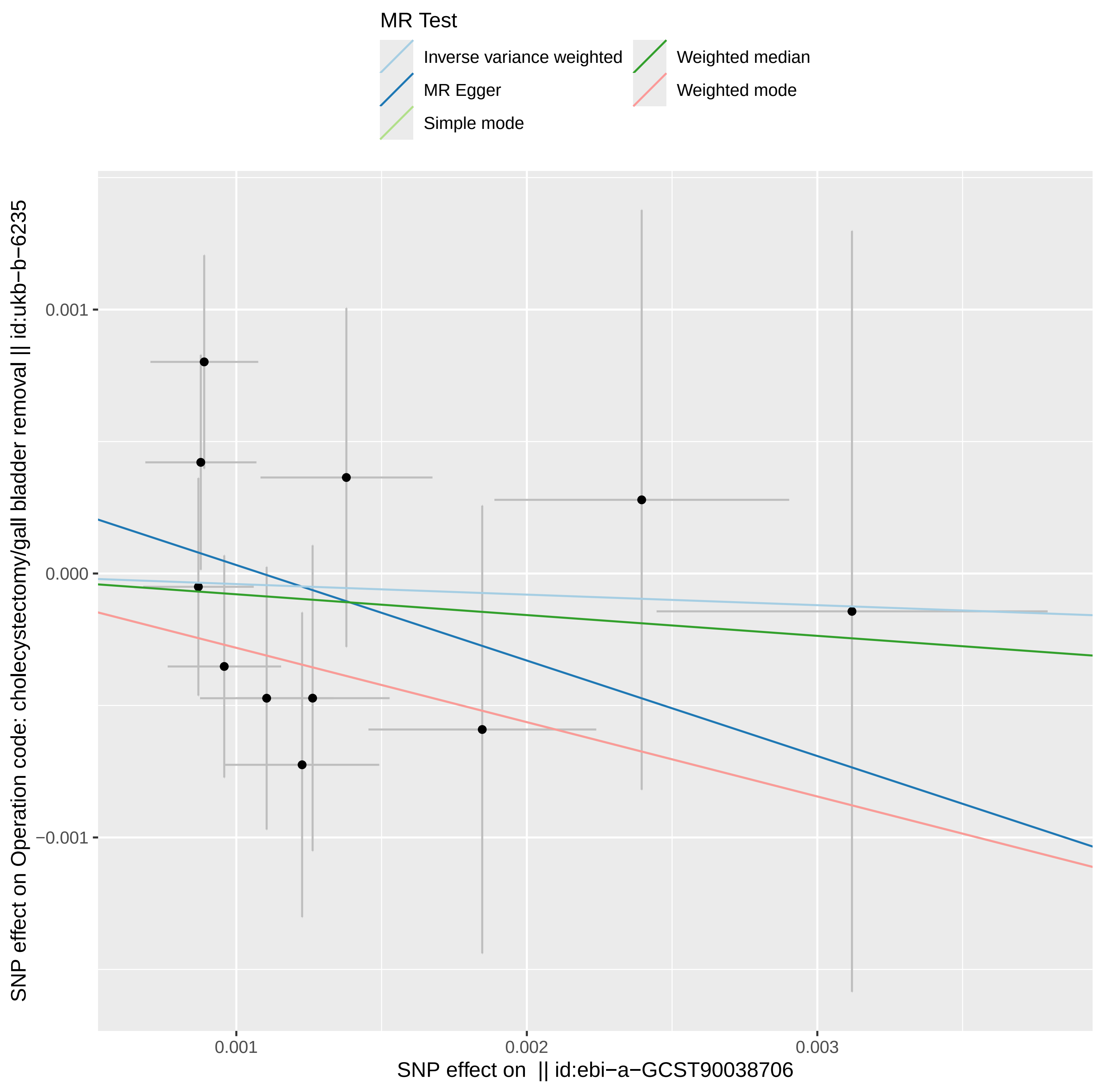
MR Test
Inverse variance weighted
MR Egger
Simple mode
Weighted median
Weighted mode
0.001
0.000
−0.001
SNP effect on Operation code: cholecystectomy/gall bladder removal || id:ukb−b−6235
0.001
0.002
0.003
SNP effect on || id:ebi−a−GCST90038706

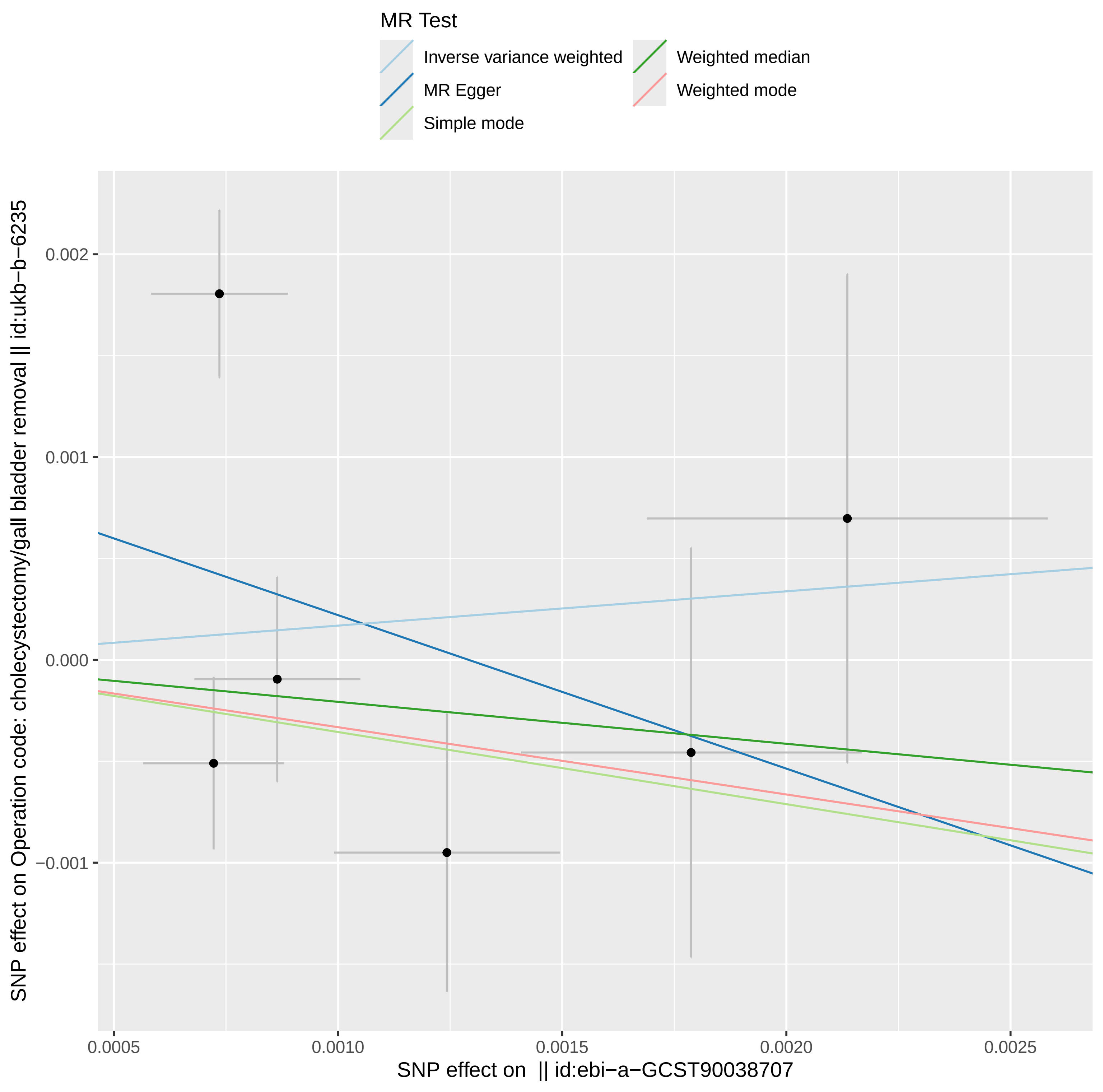

MR Test
Inverse variance weighted
MR Egger
Simple mode
Weighted median
Weighted mode
0.002
0.001
0.000
−0.001
0.0005
0.0010
0.0015
0.0020
0.0025
SNP effect on Operation code: cholecystectomy/gall bladder removal || id:ukb−b−6235
SNP effect on || id:ebi−a−GCST90038707
