# Supplementary material for: Causal association between cholecystectomy and fracture: A Mendelian randomization study
Source: Medicine (Baltimore). 2024 Dec 6;103(49):e40795. doi: 10.1097/MD.0000000000040795 (PMC11630995; doi:10.1097/MD.0000000000040795)
Supplement: Supplementary file 5 [file medi-103-e40795-s005.pdf]

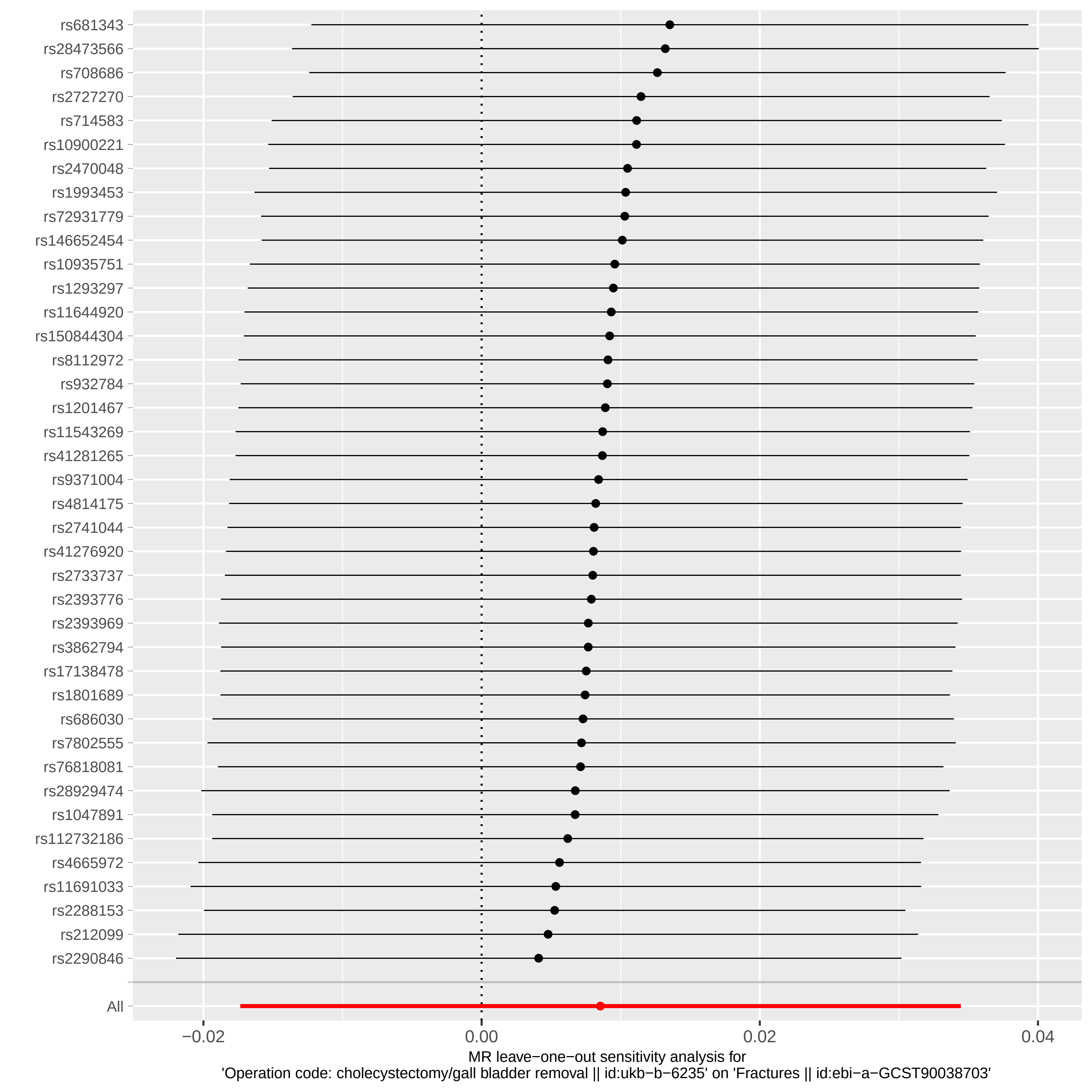

rs681343
rs28473566
rs708686
rs2727270
rs714583
rs10900221
rs2470048
rs1993453
rs72931779
rs146652454
rs10935751
rs1293297
rs11644920
rs150844304
rs8112972
rs932784
rs1201467
rs11543269
rs41281265
rs9371004
rs4814175
rs2741044
rs41276920
rs2733737
rs2393776
rs2393969
rs3862794
rs17138478
rs1801689
rs686030
rs7802555
rs76818081
rs28929474
rs1047891
rs112732186
rs4665972
rs11691033
rs2288153
rs212099
rs2290846
All
−0.02
0.00
0.02
0.04
MR leave−one−out sensitivity analysis for
'Operation code: cholecystectomy/gall bladder removal || id:ukb−b−6235' on 'Fractures || id:ebi−a−GCST90038703'

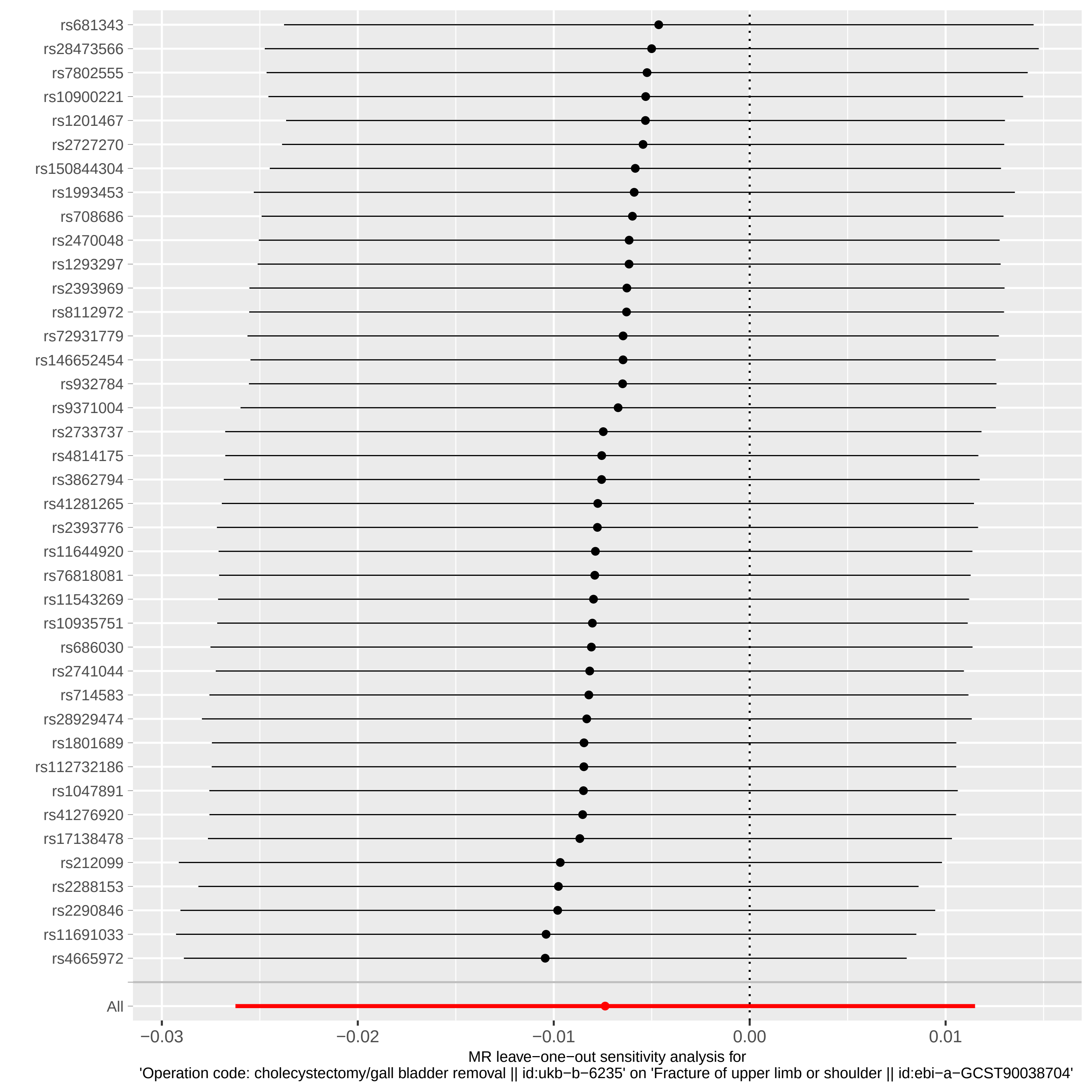
rs681343
rs28473566
rs7802555
rs10900221
rs1201467
rs2727270
rs150844304
rs1993453
rs708686
rs2470048
rs1293297
rs2393969
rs8112972
rs72931779
rs146652454
rs932784
rs9371004
rs2733737
rs4814175
rs3862794
rs41281265
rs2393776
rs11644920
rs76818081
rs11543269
rs10935751
rs686030
rs2741044
rs714583
rs28929474
rs1801689
rs112732186
rs1047891
rs41276920
rs17138478
rs212099
rs2288153
rs2290846
rs11691033
rs4665972
All
−0.03
−0.02
−0.01
0.00
0.01
MR leave−one−out sensitivity analysis for
'Operation code: cholecystectomy/gall bladder removal || id:ukb−b−6235' on 'Fracture of upper limb or shoulder || id:ebi−a−GCST90038704'

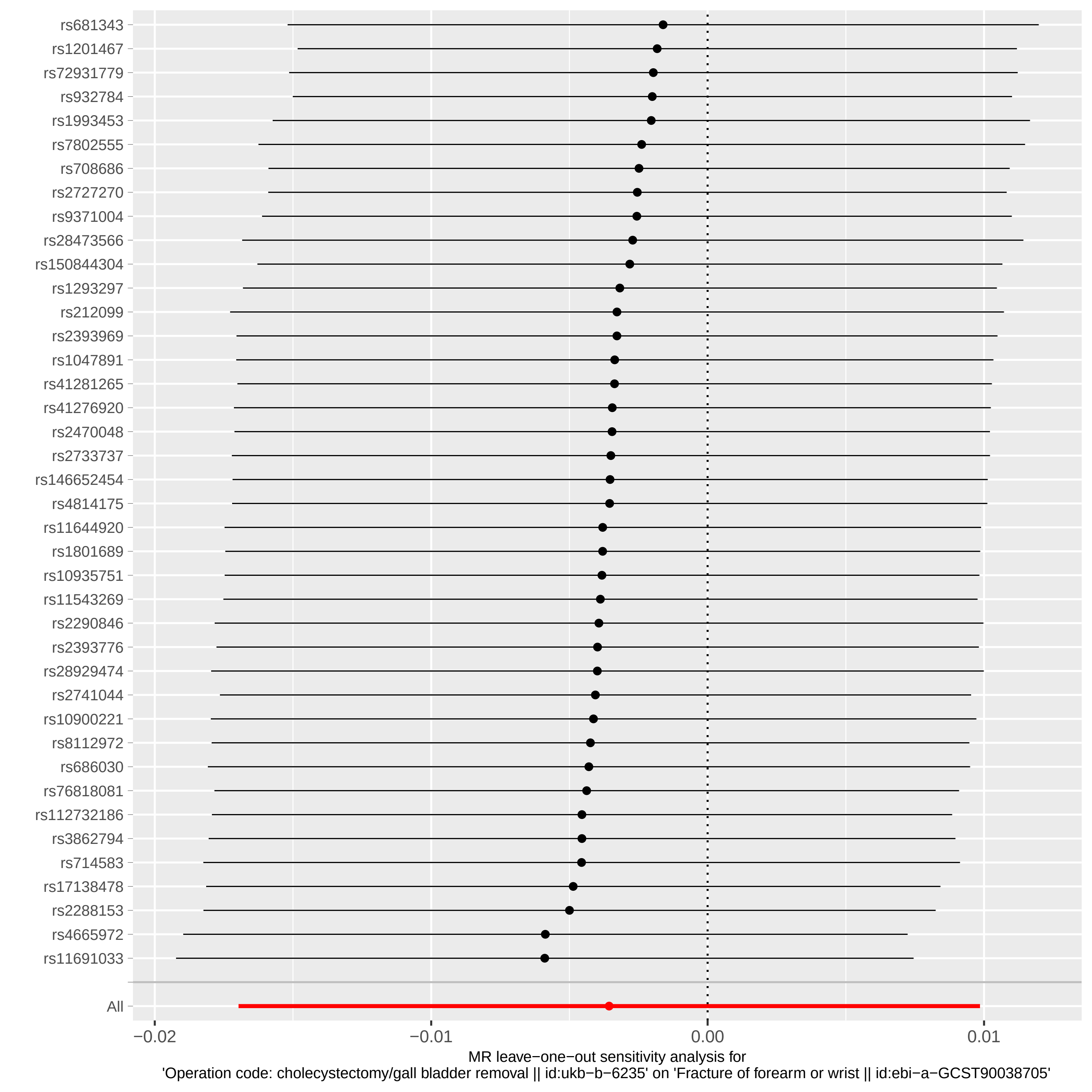

rs681343
rs1201467
rs72931779
rs932784
rs1993453
rs7802555
rs708686
rs2727270
rs9371004
rs28473566
rs150844304
rs1293297
rs212099
rs2393969
rs1047891
rs41281265
rs41276920
rs2470048
rs2733737
rs146652454
rs4814175
rs11644920
rs1801689
rs10935751
rs11543269
rs2290846
rs2393776
rs28929474
rs2741044
rs10900221
rs8112972
rs686030
rs76818081
rs112732186
rs3862794
rs714583
rs17138478
rs2288153
rs4665972
rs11691033
All
−0.02
−0.01
0.00
0.01
MR leave−one−out sensitivity analysis for
'Operation code: cholecystectomy/gall bladder removal || id:ukb−b−6235' on 'Fracture of forearm or wrist || id:ebi−a−GCST90038705'

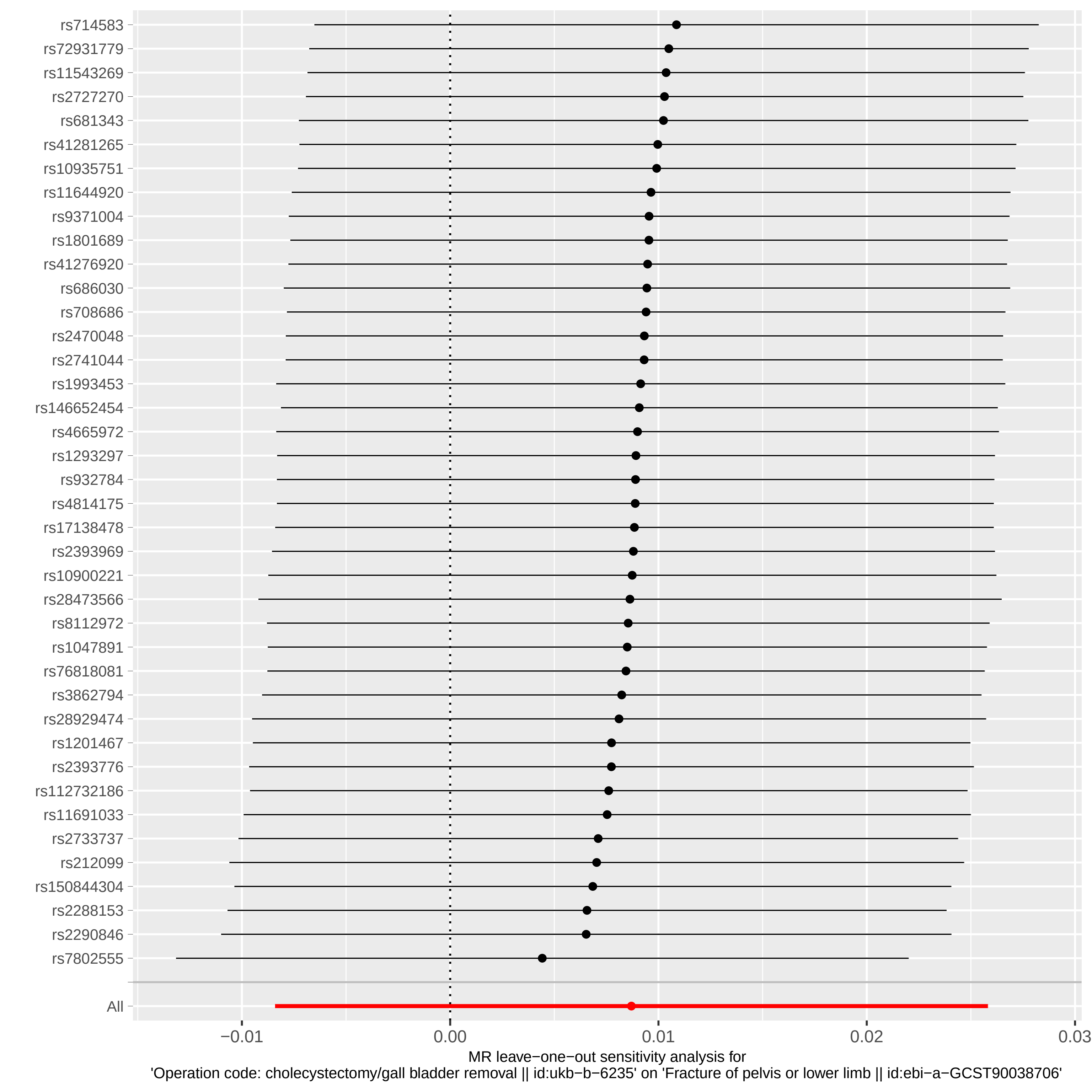

rs714583
rs72931779
rs11543269
rs2727270
rs681343
rs41281265
rs10935751
rs11644920
rs9371004
rs1801689
rs41276920
rs686030
rs708686
rs2470048
rs2741044
rs1993453
rs146652454
rs4665972
rs1293297
rs932784
rs4814175
rs17138478
rs2393969
rs10900221
rs28473566
rs8112972
rs1047891
rs76818081
rs3862794
rs28929474
rs1201467
rs2393776
rs112732186
rs11691033
rs2733737
rs212099
rs150844304
rs2288153
rs2290846
rs7802555
All
−0.01
0.00
0.01
0.02
0.03
MR leave−one−out sensitivity analysis for
'Operation code: cholecystectomy/gall bladder removal || id:ukb−b−6235' on 'Fracture of pelvis or lower limb || id:ebi−a−GCST90038706'

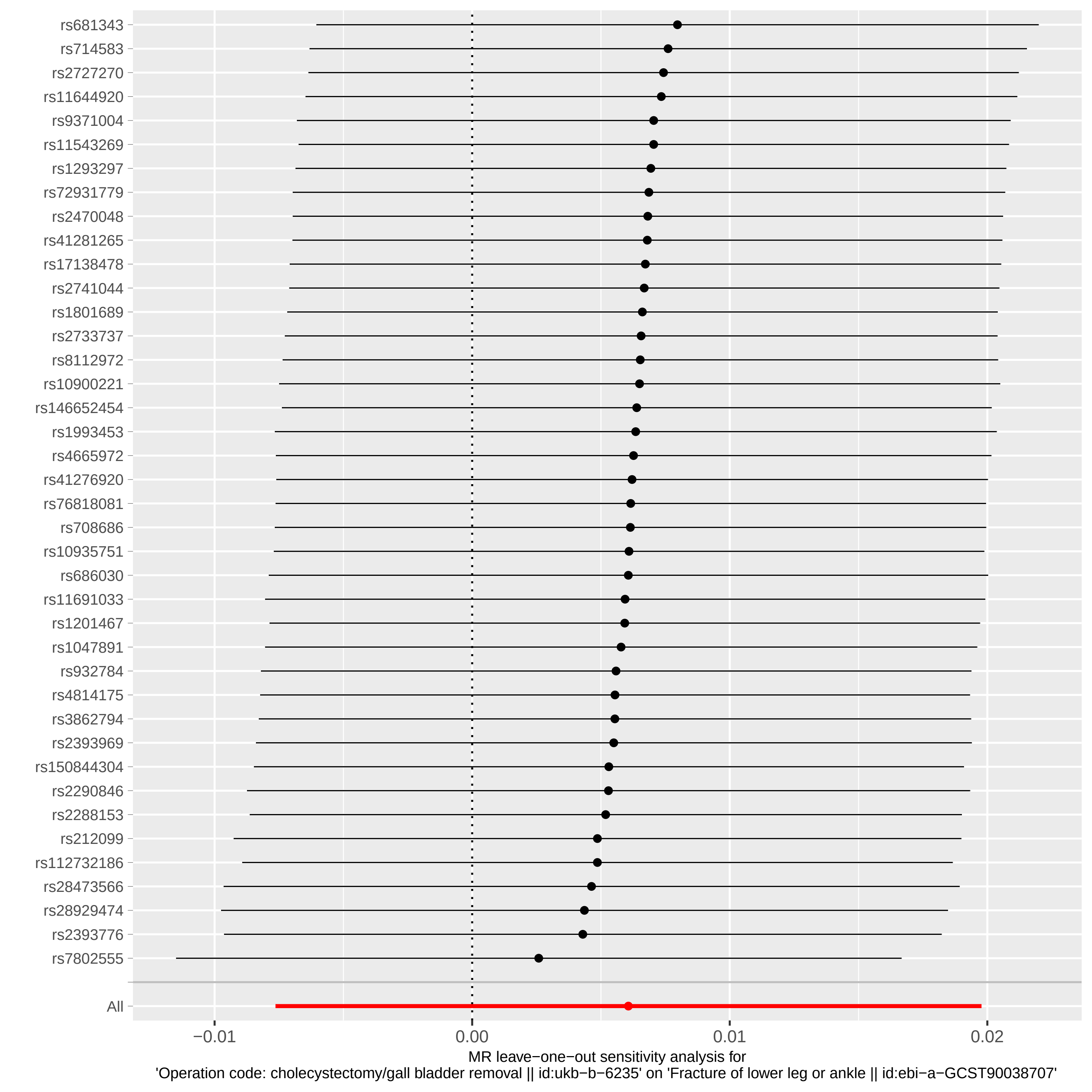
rs681343
rs714583
rs2727270
rs11644920
rs9371004
rs11543269
rs1293297
rs72931779
rs2470048
rs41281265
rs17138478
rs2741044
rs1801689
rs2733737
rs8112972
rs10900221
rs146652454
rs1993453
rs4665972
rs41276920
rs76818081
rs708686
rs10935751
rs686030
rs11691033
rs1201467
rs1047891
rs932784
rs4814175
rs3862794
rs2393969
rs150844304
rs2290846
rs2288153
rs212099
rs112732186
rs28473566
rs28929474
rs2393776
rs7802555
All
−0.01
0.00
0.01
0.02
MR leave−one−out sensitivity analysis for
'Operation code: cholecystectomy/gall bladder removal || id:ukb−b−6235' on 'Fracture of lower leg or ankle || id:ebi−a−GCST90038707'
