# Supplementary material for: Causal association between cholecystectomy and fracture: A Mendelian randomization study
Source: Medicine (Baltimore). 2024 Dec 6;103(49):e40795. doi: 10.1097/MD.0000000000040795 (PMC11630995; doi:10.1097/MD.0000000000040795)
Supplement: Supplementary file 6 [file medi-103-e40795-s006.pdf]

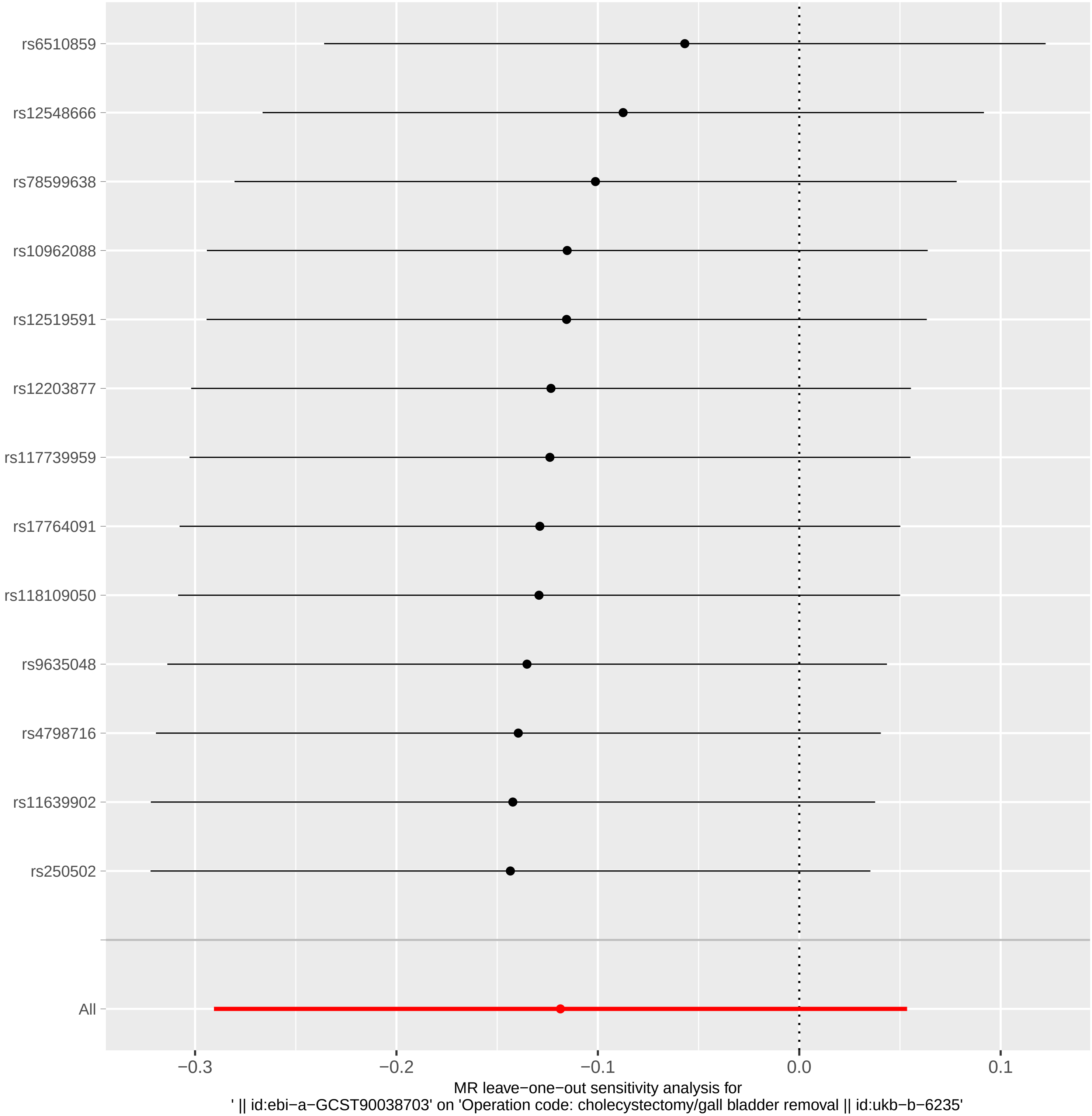

rs6510859
rs12548666
rs78599638
rs10962088
rs12519591
rs12203877
rs117739959
rs17764091
rs118109050
rs9635048
rs4798716
rs11639902
rs250502
All
−0.3
−0.2
−0.1
0.0
0.1
MR leave−one−out sensitivity analysis for
' || id:ebi−a−GCST90038703' on 'Operation code: cholecystectomy/gall bladder removal || id:ukb−b−6235'

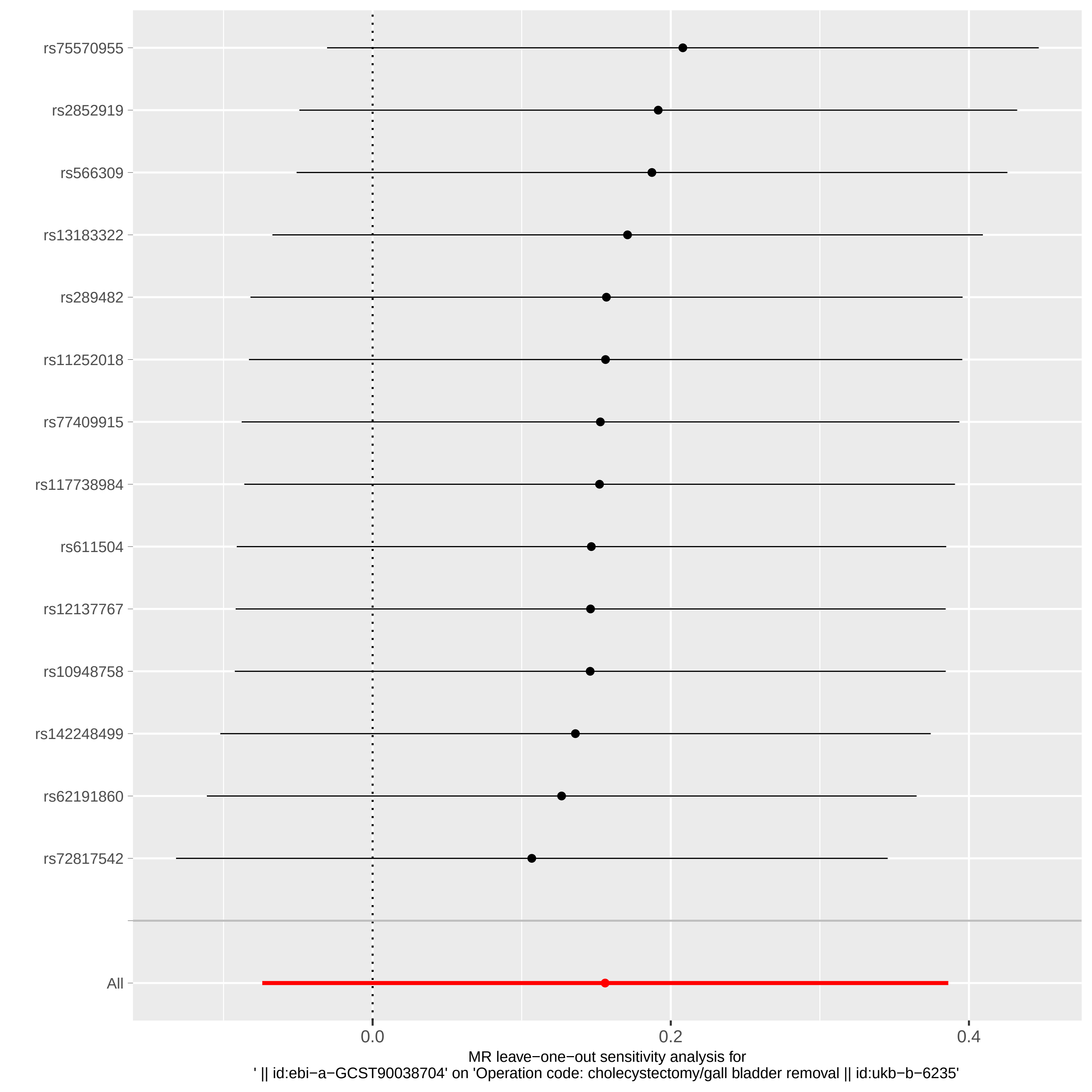
rs75570955
rs2852919
rs566309
rs13183322
rs289482
rs11252018
rs77409915
rs117738984
rs611504
rs12137767
rs10948758
rs142248499
rs62191860
rs72817542
All
0.0
0.2
0.4
MR leave−one−out sensitivity analysis for
' || id:ebi−a−GCST90038704' on 'Operation code: cholecystectomy/gall bladder removal || id:ukb−b−6235'

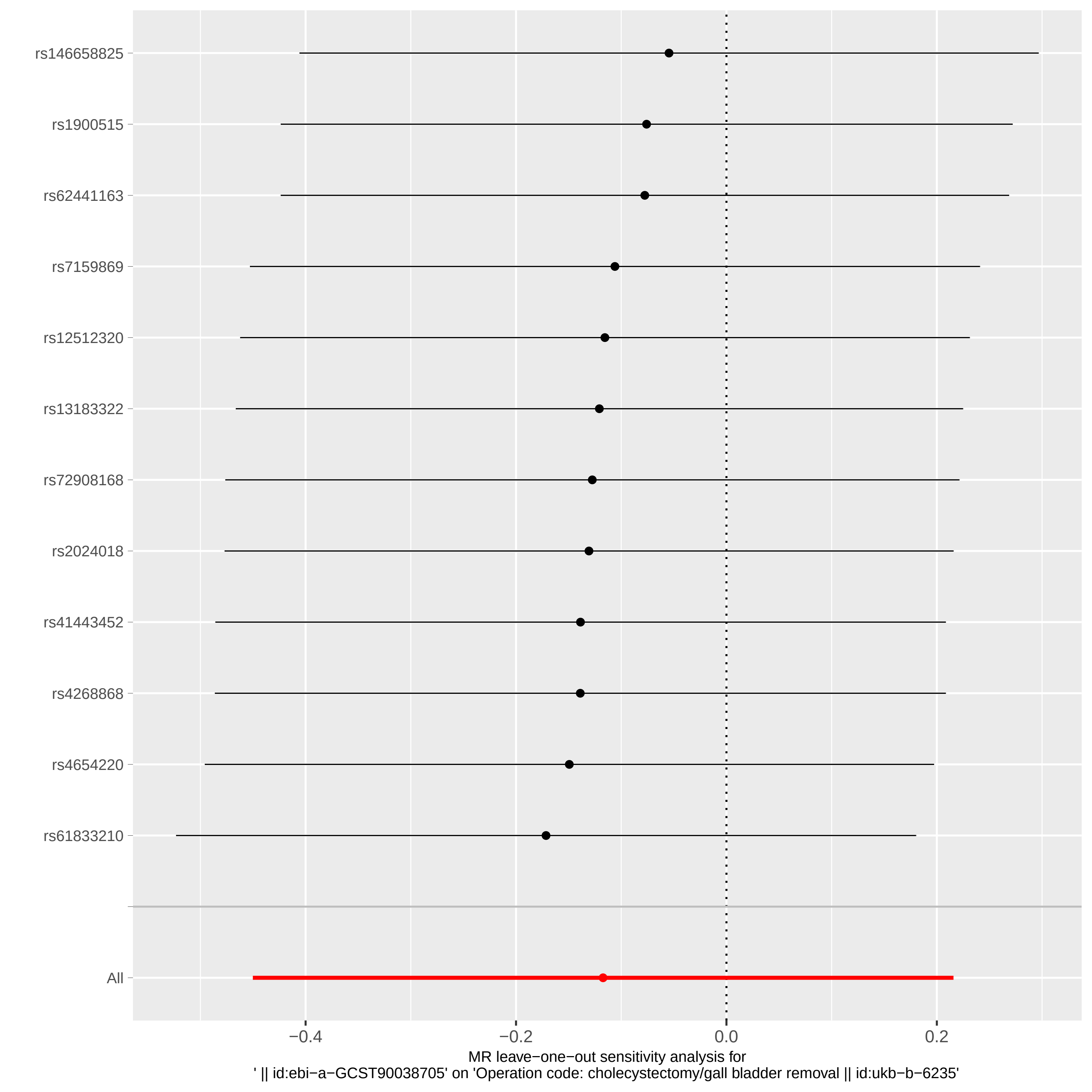

rs146658825
rs1900515
rs62441163
rs7159869
rs12512320
rs13183322
rs72908168
rs2024018
rs41443452
rs4268868
rs4654220
rs61833210
All
−0.4
−0.2
0.0
0.2
MR leave−one−out sensitivity analysis for
' || id:ebi−a−GCST90038705' on 'Operation code: cholecystectomy/gall bladder removal || id:ukb−b−6235'

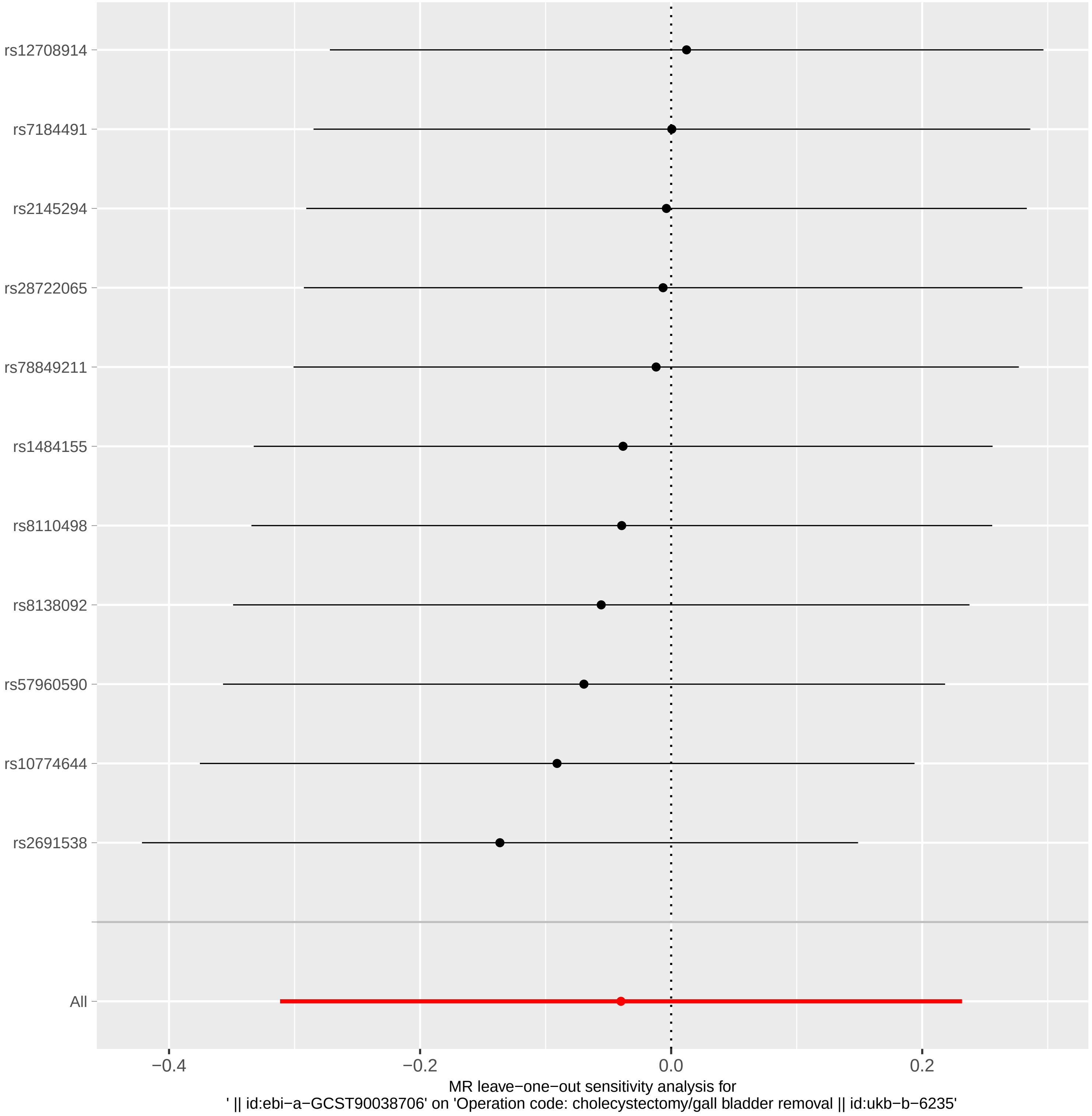
rs12708914
rs7184491
rs2145294
rs28722065
rs78849211
rs1484155
rs8110498
rs8138092
rs57960590
rs10774644
rs2691538
All
−0.4
−0.2
0.0
0.2
MR leave−one−out sensitivity analysis for
' || id:ebi−a−GCST90038706' on 'Operation code: cholecystectomy/gall bladder removal || id:ukb−b−6235'

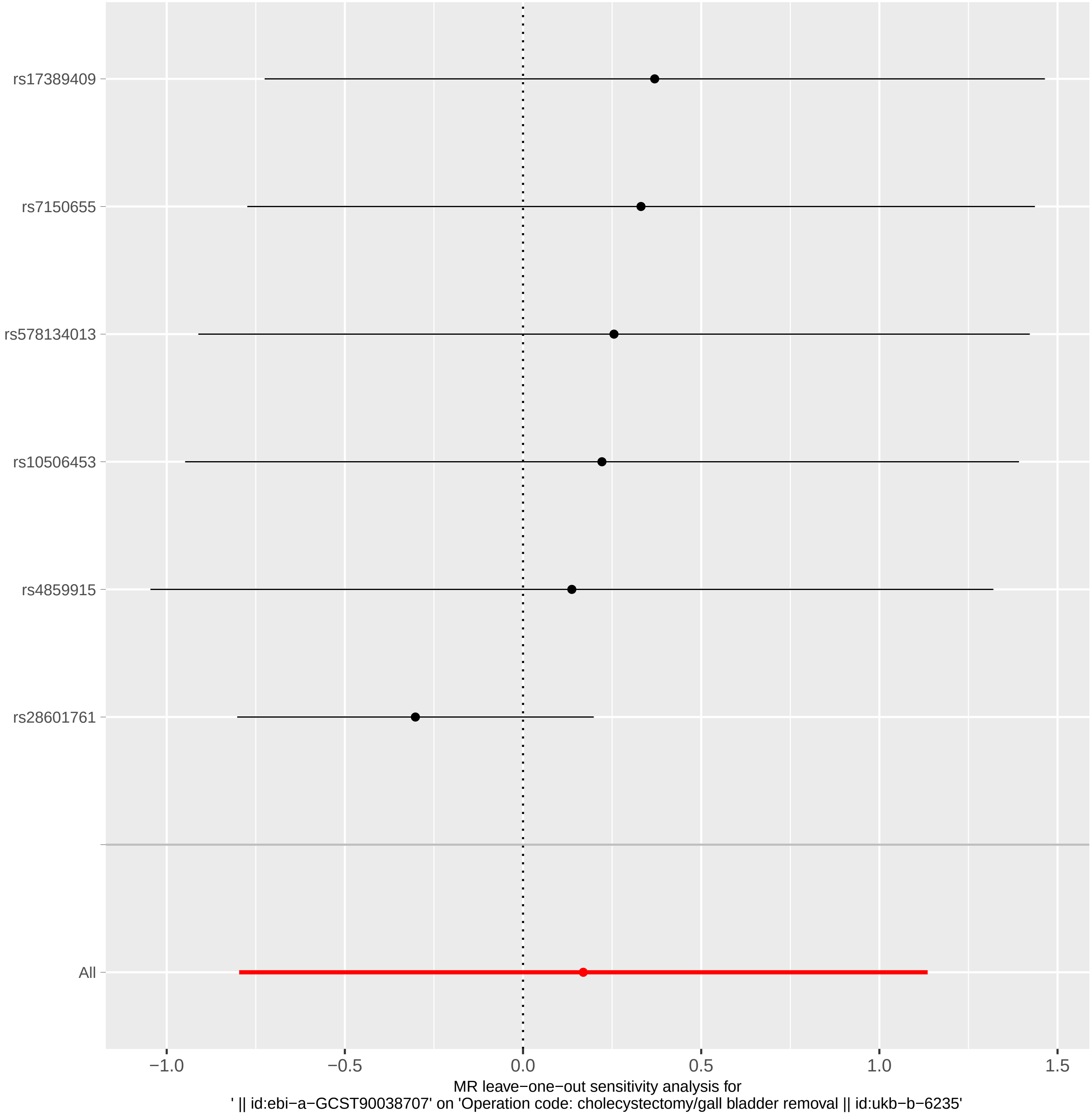

rs17389409
rs7150655
rs578134013
rs10506453
rs4859915
rs28601761
All
−1.0
−0.5
0.0
0.5
1.0
1.5
MR leave−one−out sensitivity analysis for
' || id:ebi−a−GCST90038707' on 'Operation code: cholecystectomy/gall bladder removal || id:ukb−b−6235'
