# Supplementary material for: Causal association between cholecystectomy and fracture: A Mendelian randomization study
Source: Medicine (Baltimore). 2024 Dec 6;103(49):e40795. doi: 10.1097/MD.0000000000040795 (PMC11630995; doi:10.1097/MD.0000000000040795)
Supplement: Supplementary file 7 [file medi-103-e40795-s007.pdf]

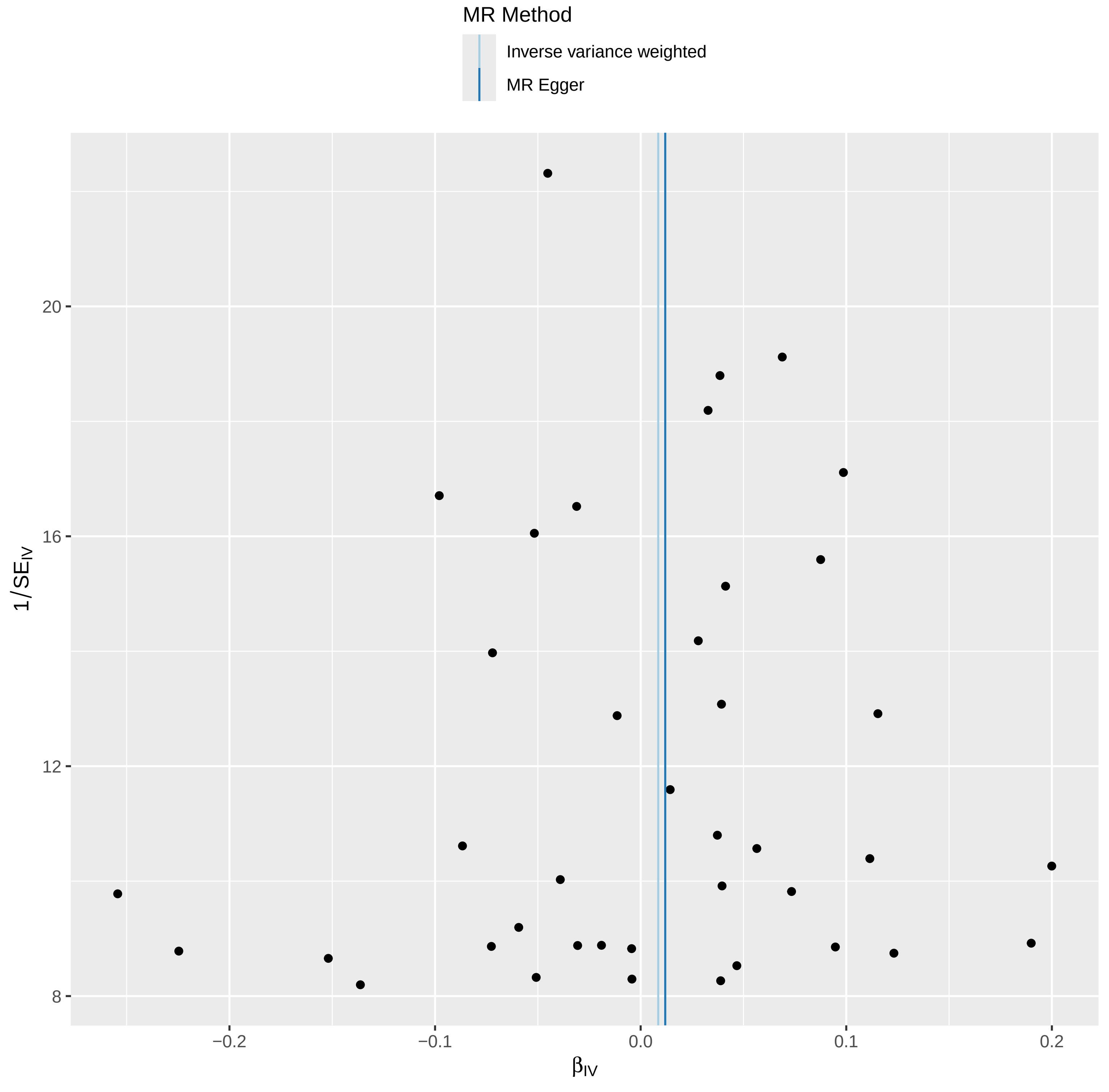

MR Method
Inverse variance weighted
MR Egger
20
16
12
8
1/SE_IV
−0.2
−0.1
0.0
0.1
0.2
β_IV

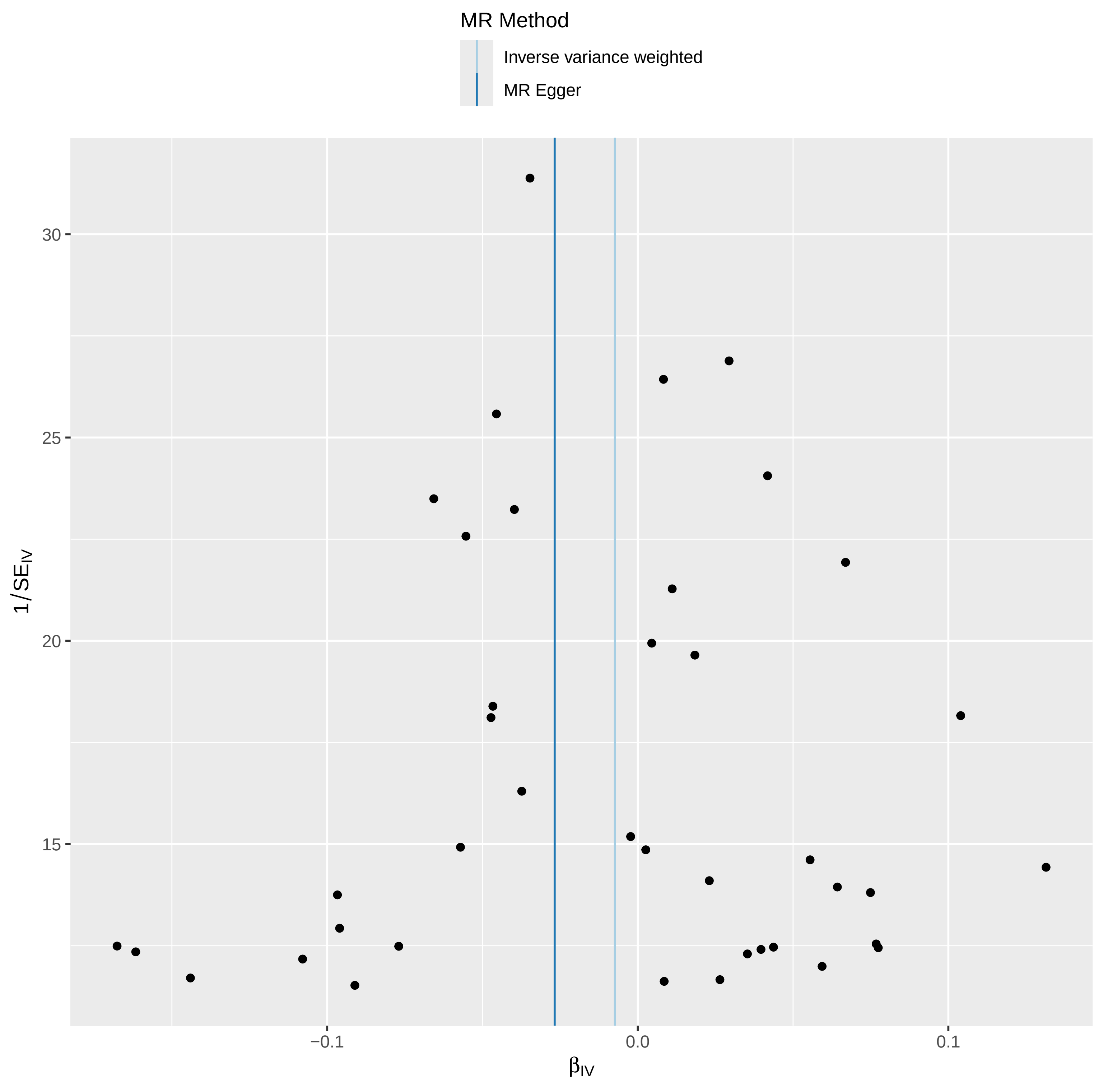
MR Method
Inverse variance weighted
MR Egger
1/SE_IV
β_IV
30
25
20
15
−0.1
0.0
0.1

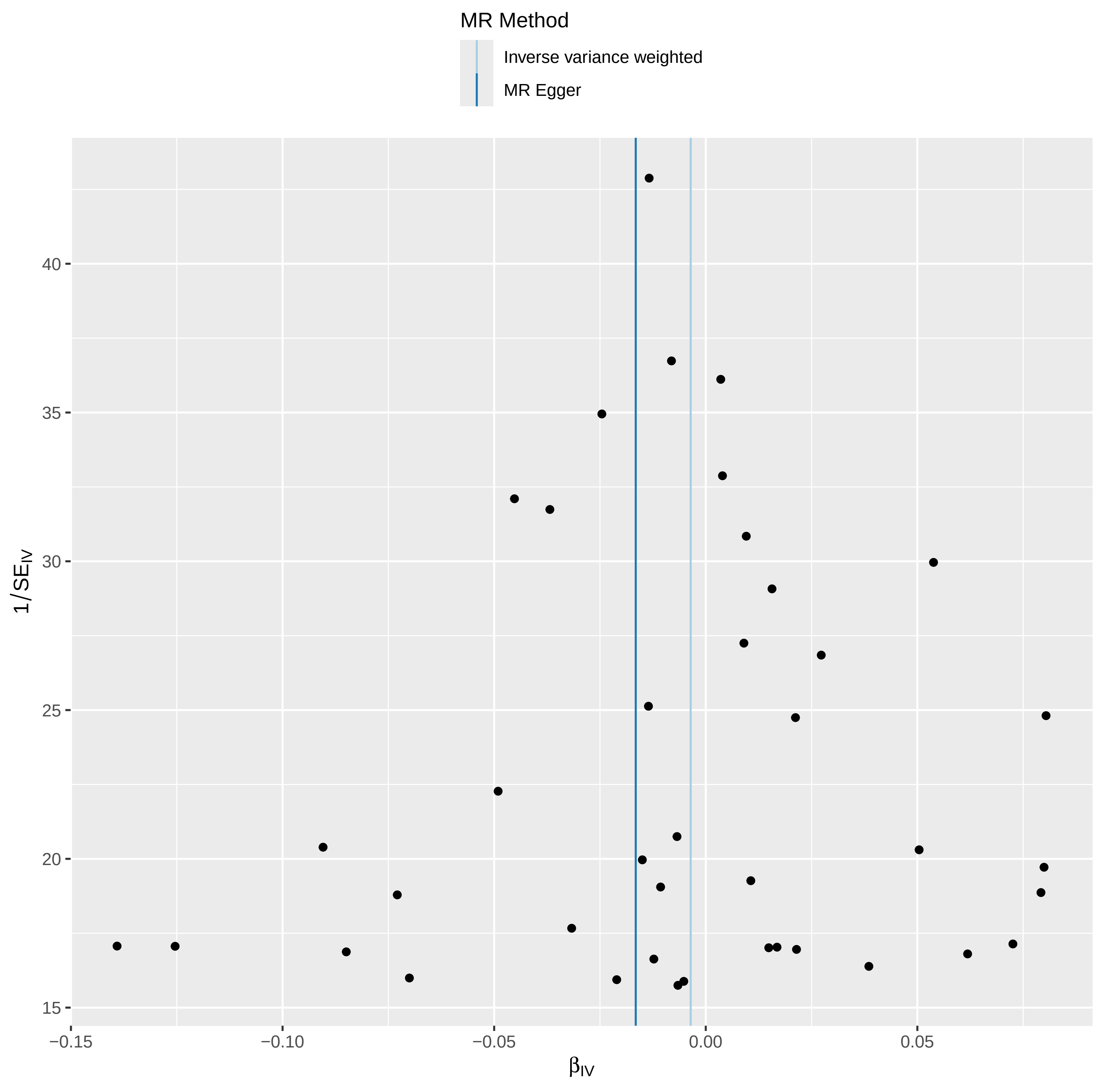
MR Method
Inverse variance weighted
MR Egger
1/SE$_{IV}$
40
35
30
25
20
15
−0.15
−0.10
−0.05
0.00
0.05
β_{IV}

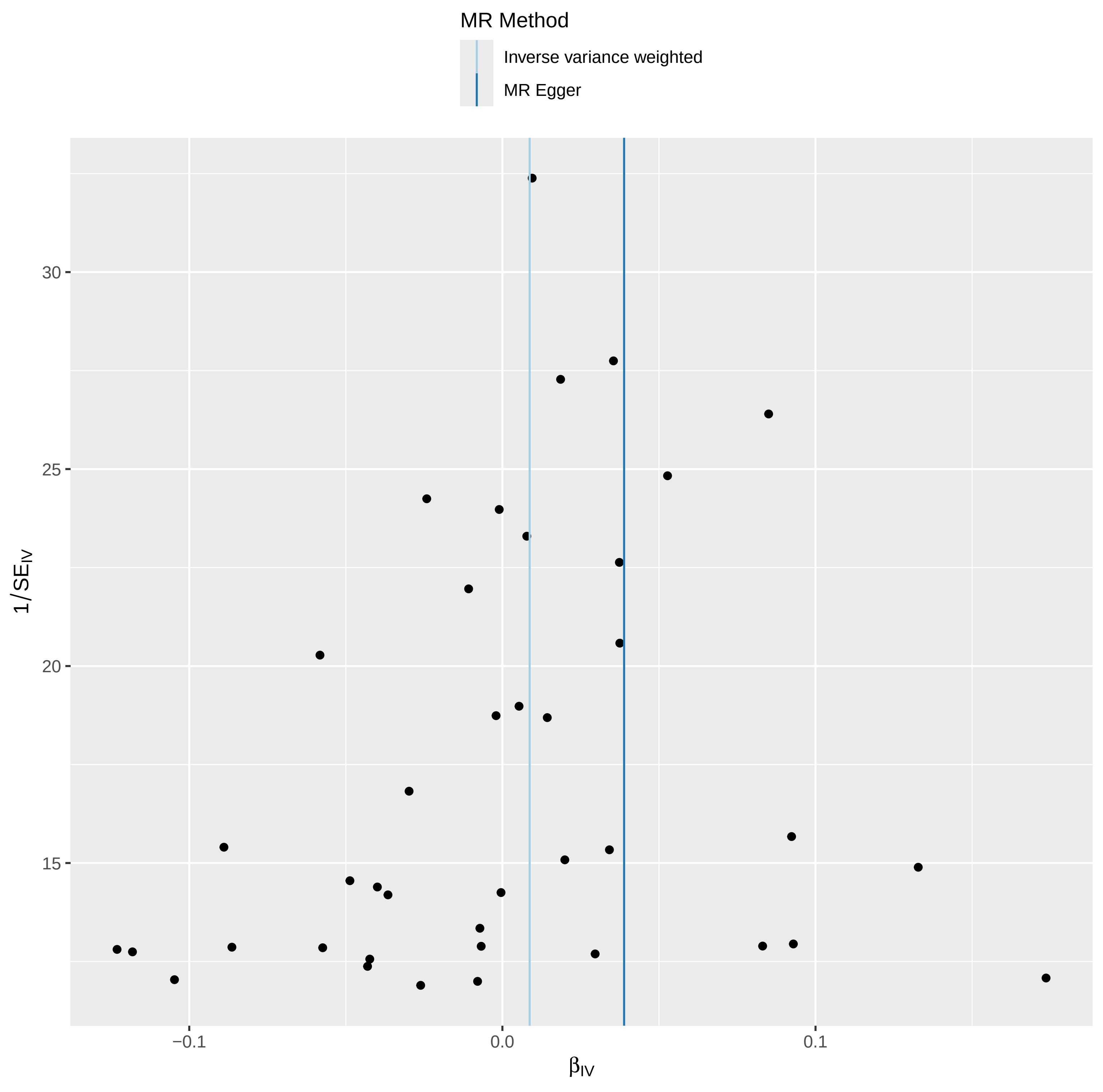
MR Method
Inverse variance weighted
MR Egger
30
25
20
15
1/SE_IV
−0.1
0.0
0.1
β_IV

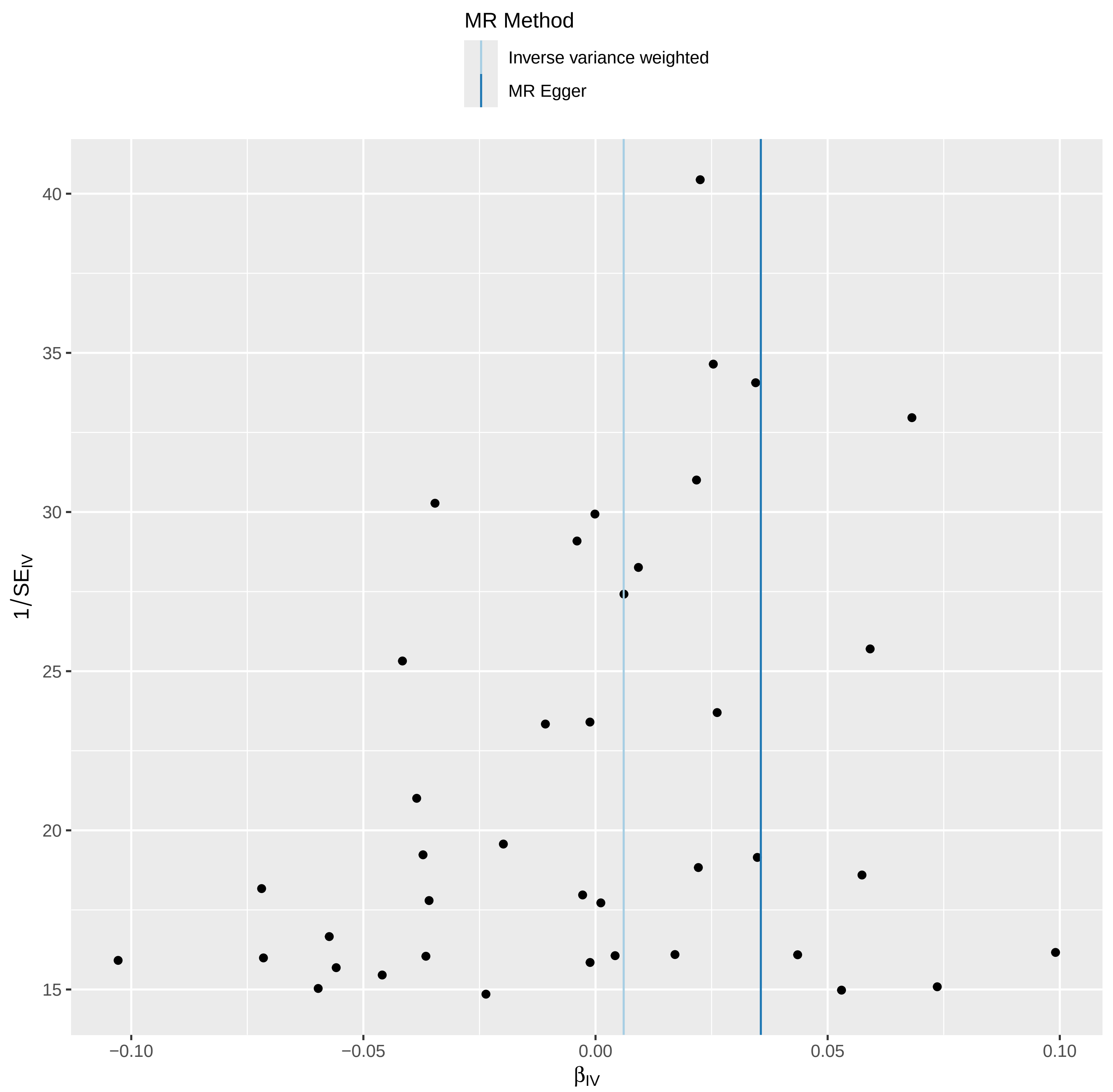
MR Method
Inverse variance weighted
MR Egger
40
35
30
25
20
15
−0.10
−0.05
0.00
0.05
0.10
$1/SE_{IV}$
β_{IV}
