# Supplementary material for: Causal association between cholecystectomy and fracture: A Mendelian randomization study
Source: Medicine (Baltimore). 2024 Dec 6;103(49):e40795. doi: 10.1097/MD.0000000000040795 (PMC11630995; doi:10.1097/MD.0000000000040795)
Supplement: Supplementary file 8 [file medi-103-e40795-s008.pdf]

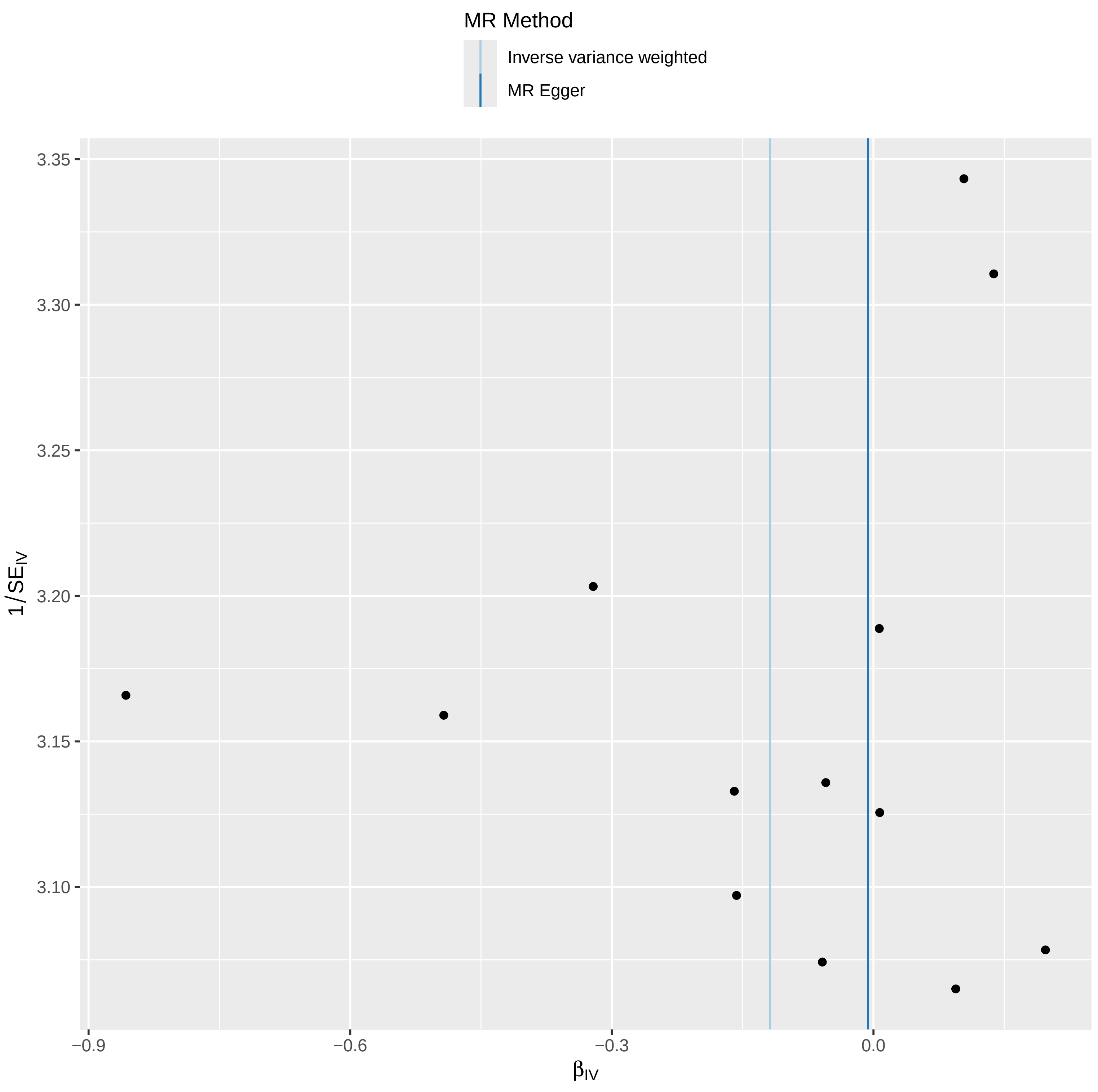
MR Method
Inverse variance weighted
MR Egger
3.35
3.30
3.25
3.20
3.15
3.10
$1/SE_{IV}$
−0.9
−0.6
−0.3
0.0
β_{IV}

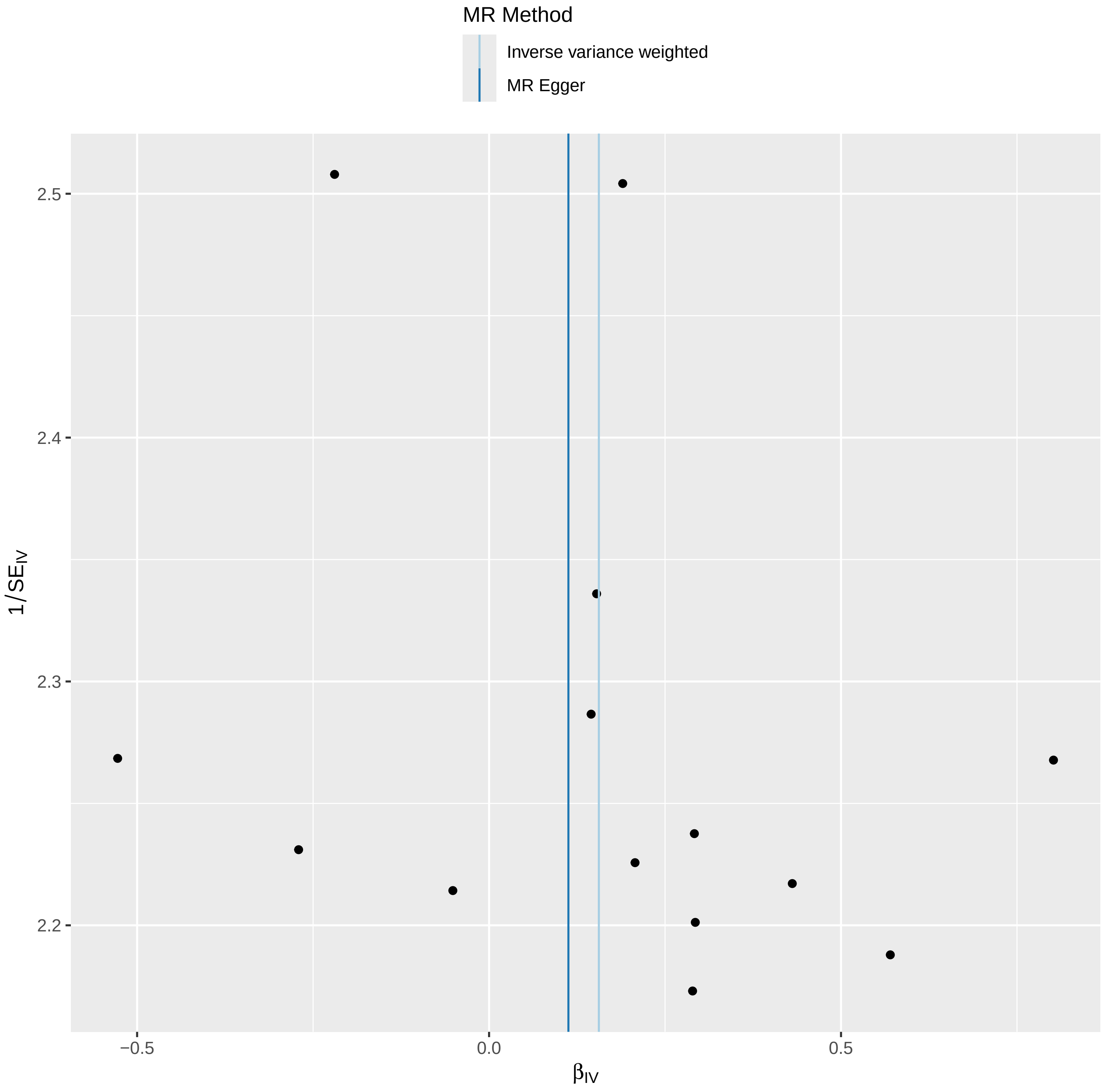

MR Method
Inverse variance weighted
MR Egger
2.5
2.4
2.3
2.2
$1/SE_{IV}$
−0.5
0.0
0.5
β_{IV}

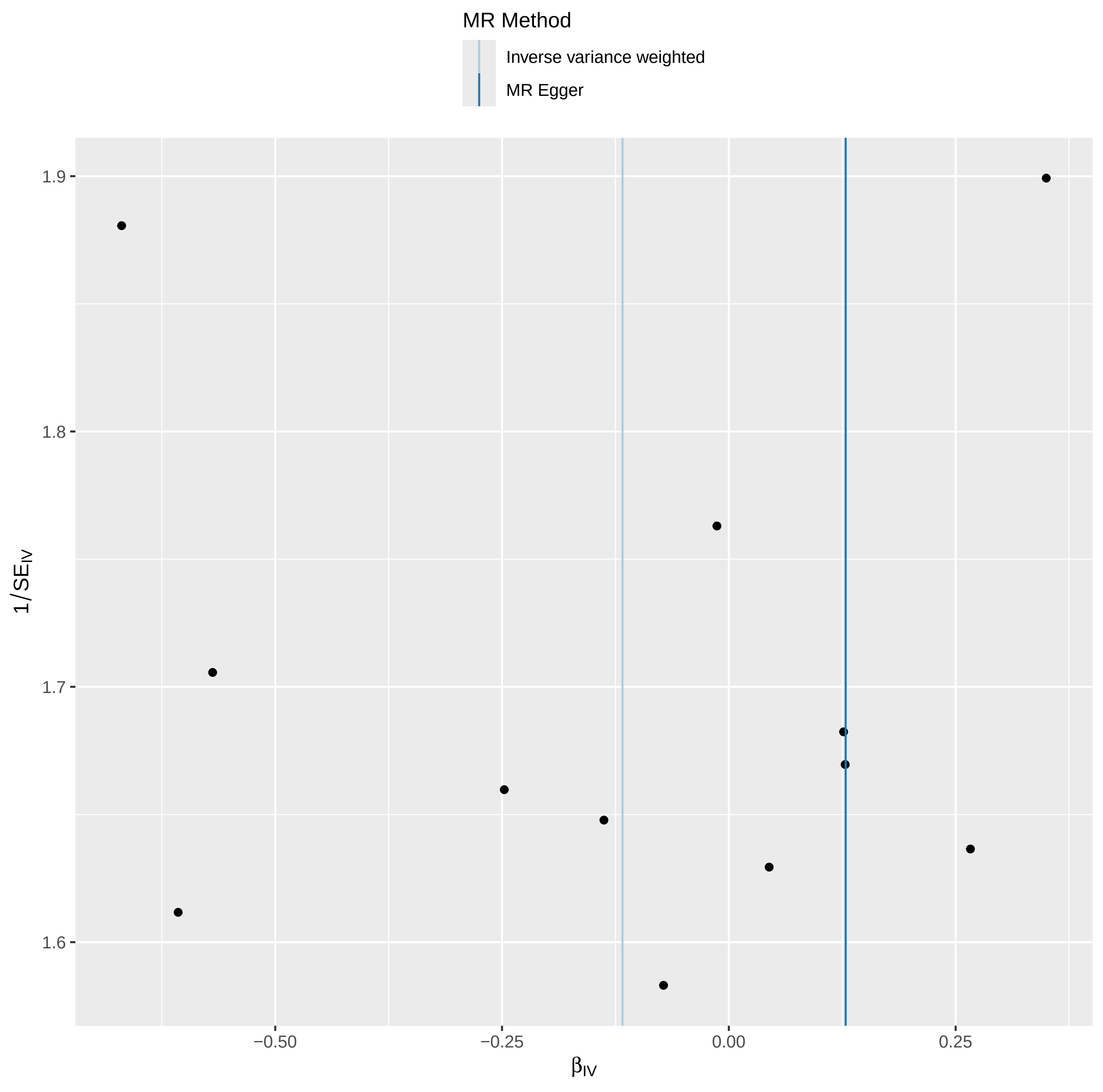

MR Method
Inverse variance weighted
MR Egger
1.9
1.8
1.7
1.6
−0.50
−0.25
0.00
0.25
$1/SE_{IV}$
β_{IV}

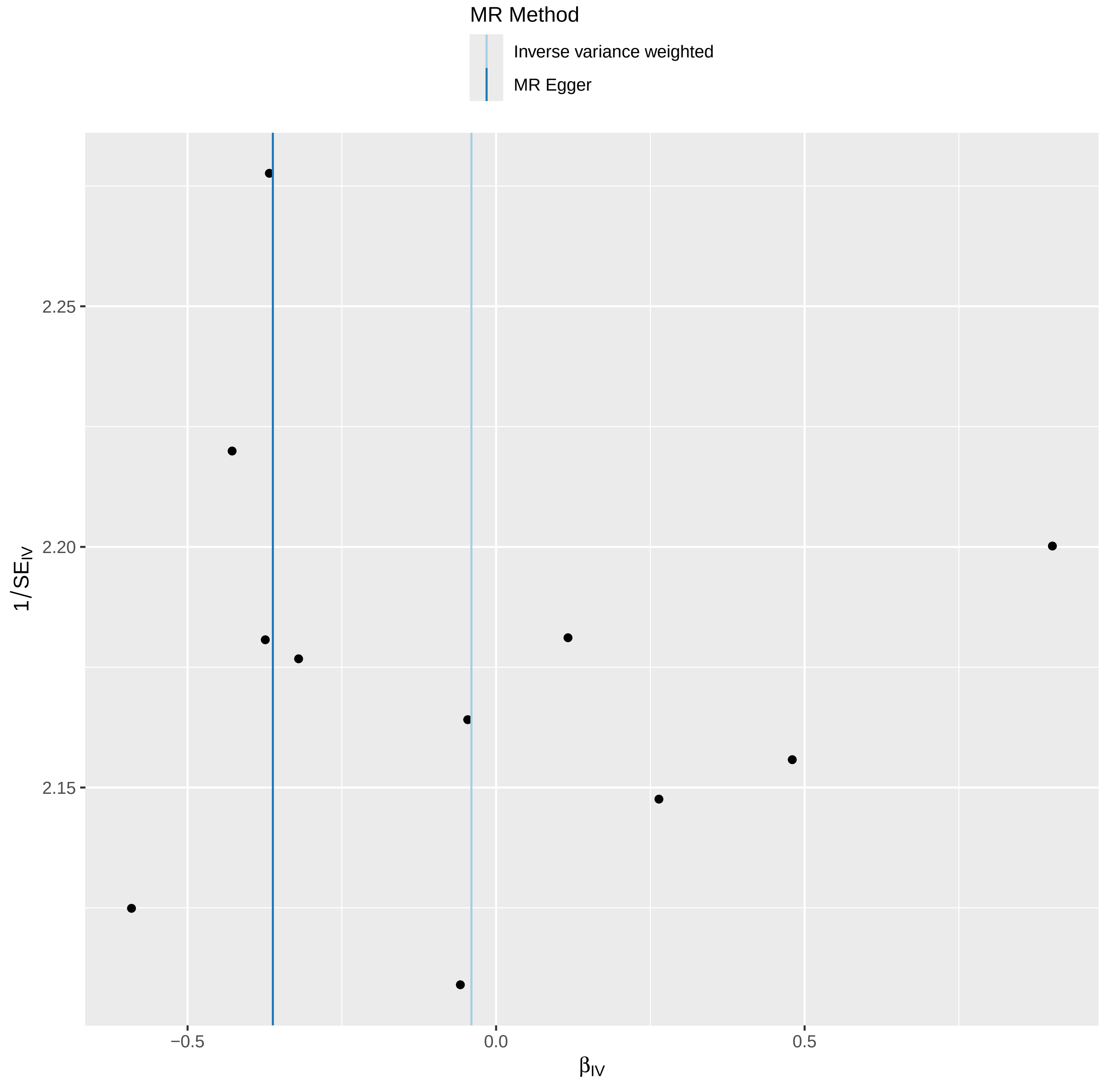
MR Method
Inverse variance weighted
MR Egger
2.25
2.20
2.15
1/SE$_{IV}$
−0.5
0.0
0.5
β$_{IV}$

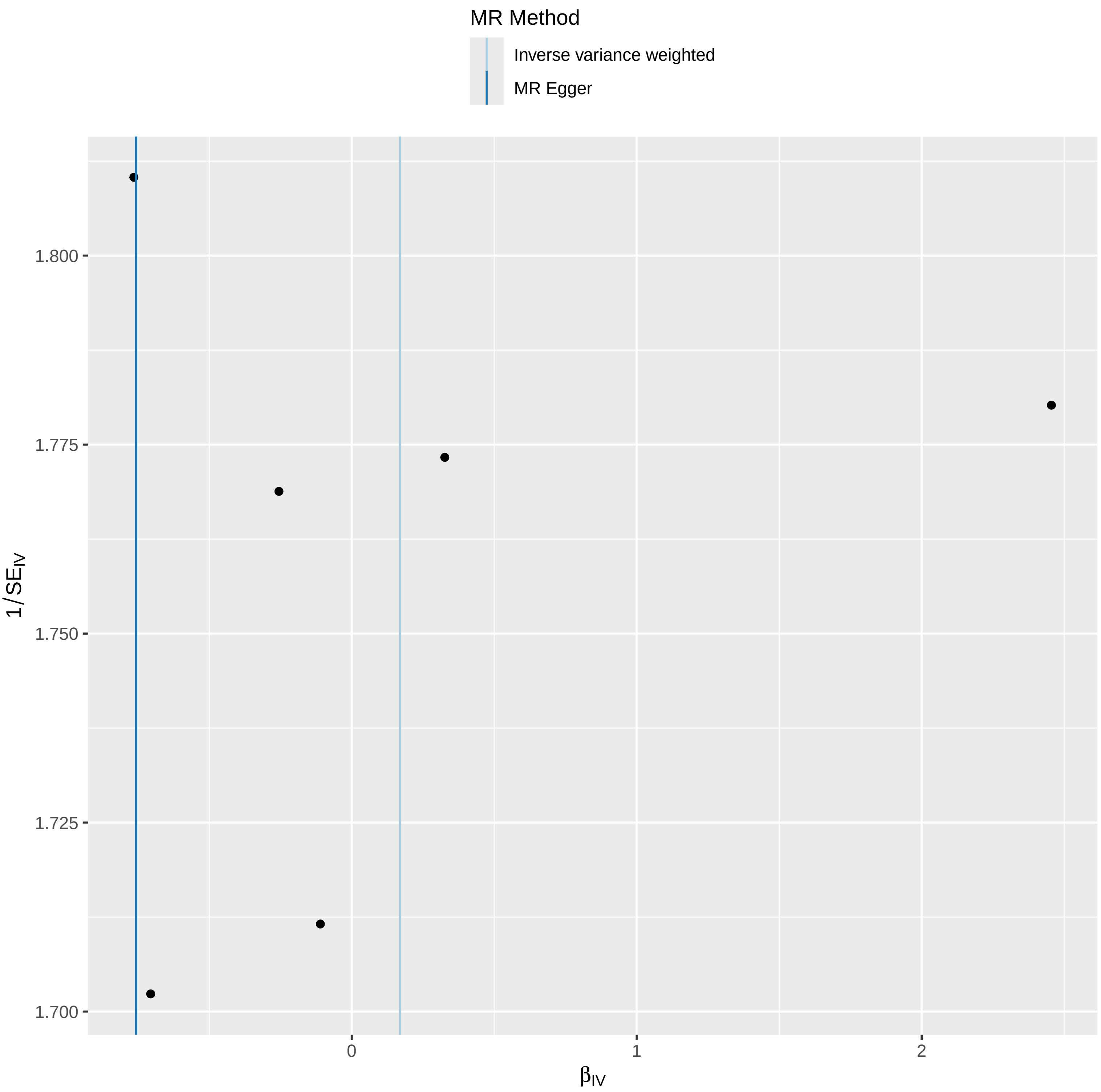
MR Method
Inverse variance weighted
MR Egger
1.800
1.775
1.750
1.725
1.700
1/SE_IV
0
1
2
β_IV
